# Clinical Landscape of Littoral Cell Angioma in the Spleen Based on a Comprehensive Analysis

**DOI:** 10.3389/fonc.2022.790332

**Published:** 2022-02-08

**Authors:** Weijie Wang, Guangzhao Qi, Xiangtian Zhao, Yanping Zhang, Rongtao Zhu, Ruopeng Liang, Yuling Sun

**Affiliations:** ^1^ Department of Hepatobiliary and Pancreatic Surgery, The First Affiliated Hospital of Zhengzhou University, Zhengzhou, China; ^2^ Department of Pharmacy, The First Affiliated Hospital of Zhengzhou University, Zhengzhou, China; ^3^ Department of Radiology, Guangdong Provincial People’s Hospital, Guangdong Academy of Medical Sciences, Guangzhou, China; ^4^ Department of Pathology, The First Affiliated Hospital of Zhengzhou University, Zhengzhou, China

**Keywords:** littoral cell angioma, diagnosis, treatment, prognosis, systematic review

## Abstract

**Objective:**

Littoral cell angioma (LCA) is currently considered to be a rare splenic tumor with malignant potential. As the epidemiology, pathogenesis, clinical manifestation, treatment, and prognosis remain unclear, the clinical diagnosis and treatment of LCA have not been standardized. Hence, we performed a comprehensive analysis of 189 observational studies comprising 435 patients to improve the current status of diagnosis and treatment.

**Methods:**

PubMed, Embase, WanFang and CNKI were searched from inception to May 2021 to identify LCA studies that were published in English and Chinese. The clinical information of LCA patients were extracted and analyzed.

**Results:**

The LCA has a male-to-female ratio of 0.90 and a solitary-to-multiple ratio of 0.31. In terms of clinical features, 69.7% of the patients showed splenomegaly, 49.7% were asymptomatic, and 39.2% experienced epigastric discomfort. As the imaging findings of patients with LCA were nonspecific, an image-guided biopsy (10/12) was a safe and effective method for diagnosing in this condition. Notably, results of the prognostic analysis indicated that LCA has a lower risk of recurrence and metastasis. The patient may develop a stable disease or the tumor will grow but will not metastasize. Besides, the novel immunohistochemical pattern of LCA was described as CD31^+^/ERG^+^/FVIII Antigen^+^/CD68^+^/CD163^+^/lysozyme^+^/CD8^−^/WT1^−^.

**Conclusion:**

LCA should be reconsidered as a benign primary splenic vascular neoplasm, which is more like an intra-splenic manifestation of abnormal body function. Image-guided biopsy with follow-up might be a beneficial choice for LCA patients. For LCA patients with abdominal discomfort, pathological uncertainty or continuous tumor enlargement, splenectomy remains the preferred treatment.

## Introduction

Littoral cell angioma (LCA) is a rare splenic vascular neoplasm originating from the littoral cell lining the red sinuses (1–4). Falk was the first to describe and name this entity; since its initial description, more and more studies have been conducted to investigate LCA (1, 5–9). Due to the extremely low incidence and a lack of awareness of LCA, all studies were conducted as case reports and only included less than 27 samples; moreover, nearly all the cases were misdiagnosed as other diseases (10–12). The diagnosis of LCA was primarily based on the pathological findings (1, 12, 13). In fact, littoral cell tumors (LCT) could be divided into three types with the same immunophenotype, namely, LCA, littoral cell hemangioendothelioma (LCHE), and littoral cell angiosarcoma (LCAS) (14). Because the latter two exhibited a typical nuclear atypia and malignant biological behavior, they were considered to have an intermediate malignant potential and malignant, respectively. Although LCA was considered as benign by clinicians, it was closely related to a variety of malignant tumors (14). Moreover, some patients with LCA developed recurrence and distant metastasis (11, 15, 16). Thus, LCA has always been recognized as a primary tumor of the spleen with a malignant potential.

Currently, the majority of LCA patients who underwent splenectomy were at the risk of long-term complications such as sepsis, thrombus and tumor (17, 18). As little is known about the epidemiology, natural history, pathogenesis, clinical manifestations, treatment, and prognosis of LCA, an objective evaluation of all LCA cases reported is a pressing need with important clinical significance. However, systematic and quantitative assessments of the published findings of LCA patients have not been conducted. Therefore, we aimed to perform a systematic review and analysis of all LCA cases to summarize its characteristics and to explore a new mode of diagnosis and treatment.

## Materials and Methods

### Data Sources and Search Strategy

Two researchers independently searched the PubMed, Embase, WanFang, and CNKI databases from the inception to May 2021 to find published articles related to LCA. The search terms used included littoral cell angioma or littoral cell tumor or littoral cell without restrictions. Furthermore, the reference lists of the retrieved studies and recent reviews were reviewed to identify additional potentially relevant articles. This study was approved by the Institutional Review Board of the First Affiliated Hospital of Zhengzhou University (2017-xy-002). The typical imaging and pathological pictures presented in the figures were obtained from our center.

### Data Extraction

Two investigators independently evaluated all records by screening the title and abstract for potentially eligible studies, and differences in opinion were resolved by consensus with a third reviewer. Studies (i) in which a postoperative pathology confirmed the diagnosis of LCA; (ii) that reported the epidemiology, natural history, pathogenesis, clinical manifestations, treatment and prognosis of LCA; (iii) that were published in English or Chinese before May 2021; were included in the analysis. However, reviews and LCA patients repeatedly published by the same unit were excluded. The clinical information on the epidemiology, pathogenesis, clinical manifestation, treatment, and prognosis of LCA patients were extracted and analyzed.

### Statistical Analyses

The graph was drawn using Graph Pad statistics software (Graph Pad Software Inc, USA). The statistical indexes were expressed as mean and standard deviation with enumeration data, which were consistent with the normal distribution. Otherwise, it was expressed as median and interquartile range (IQR).

## Results

### General Characteristics of the LCA Population

**Figure 1:**
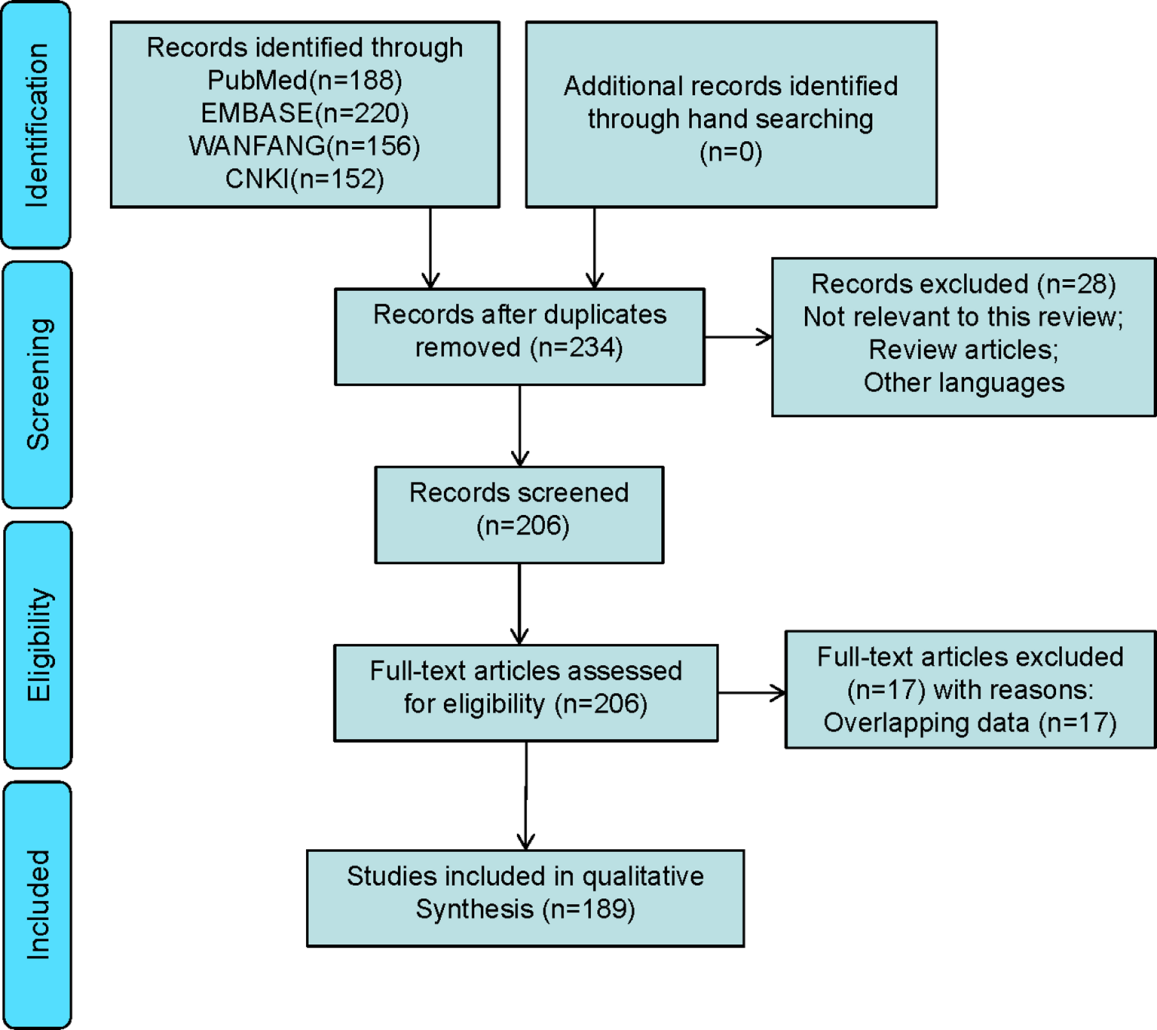
Selection of studies for inclusion in the systematic review of LCA.

**Figure 2:**
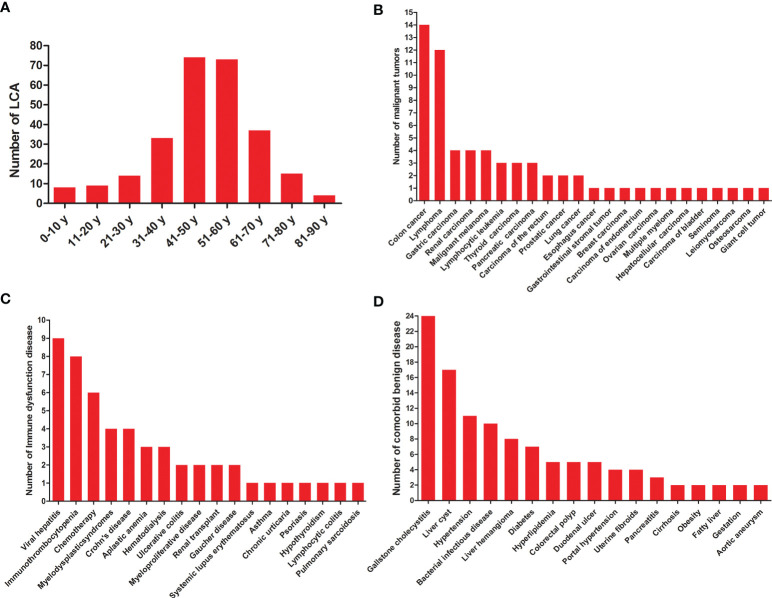
General characteristics of LCA patients. **(A)** Age distribution of LCA. **(B)** Statistics on various malignant tumors co-occurred with LCA. **(C)** Statistics on comorbid diseases of immune dysfunction. **(D)** Statistics on comorbid benign diseases except for immune dysfunction.

A total of 189 studies comprising 435 patients met the inclusion criteria (**Figure 1**, **Table 1**, and **Table S1**), namely, 171 foreigners and 264 Chinese individuals. The statistical analysis showed a male-to-female ratio of 0.90 in 405 patients with LCA, which indicated the absence of gender predilection. The average age of 267 patients was 48.4 ± 16 years, with the oldest and youngest being 86 years old and 26 days old, respectively (11, 19). As shown in **Figure 2**, LCA was more prevalent in the 40–60-year age group; however, it was detected in 15 minors (1, 19–30).

**Table 1:** General characteristics of LCA population.

General characteristics	Value	N. With data reported
Total number of LCA patients	435	171(English)/264(Chinese)
Male/Female	0.90	192/213
Age (mean, SD)	48.2(16)	267
Age range	26 d–86 y	267
Comorbid malignant tumors	13.8%	60/435^*^
The diagnosis of LCA after malignant tumors	98.3%	59/60
Comorbid diseases of immune dysfunction	12.2%	53/435

^*^One patient successively developed three kinds of malignant tumors. Three patients developed two types of malignant tumors. We focused on the first malignancy and calculated the comorbid incidence.

A total of 60 various malignant tumors co-occurred with LCA (1, 11, 12, 31–65), as shown in **Table 1** and **Figure 2**, with a comorbidity rate of 13.8%. A total of 59 patients had comorbid malignancies, which occurred before the formation of LCA. Only one patient died of gastric cancer 2 years after undergoing splenectomy (53). Considering the typical survival time of gastric cancer, we speculated that this LCA patient was very likely to have developed gastric cancer at or before the time of splenectomy. Moreover, 53 immune disorders occurred along with LCA (1, 9, 11, 24, 27, 34, 35, 39, 44, 48, 50, 52, 66–88), with a comorbidity rate of 12.2%. It is worth noting that 9 patients had both malignant tumors and immune dysfunctions prior to the development of LCA. In addition, we summarized the different benign comorbidities reported for reference by other studies (**Figure 2**).

### Quantification and Analysis of the Clinical Manifestations and Prognosis

A total of 314 patients were described with symptoms, of whom 49.7% were asymptomatic or accidental findings, 39.2% experienced upper abdominal discomfort, and 5.4% developed fever, as shown in **Table 2**. The spleen size was measured in 366 LCA patients, and 69.7% of them showed splenomegaly. Approximately 48.8% (127/260) of the patients had thrombocytopenia, while 31.4% (69/220) had anemia. Given its malignant potential, splenectomy with long-term follow up is the recommended treatment for LCA. Of the 401 patients, 81.0 and 19.0% underwent open and laparoscopic splenectomy, respectively. Although the accurate diagnosis rate was only 83.3% (10/12), image-guided biopsy was a relatively safe method when spleen lesions were difficult to diagnose.

**Table 2:** Quantification and analysis of the clinical manifestations and prognosis.

Clinical data	Value	N. With data reported
**Clinical manifestations**		
Asymptomatic or incidental finding	49.7%	156/314^*^
Upper abdominal discomfort^$^	39.2%	123/314
Fever	5.4%	17/314
Fatigue	8.3%	26/314
Dizziness	2.9%	9/314
Purpura	2.2%	7/314
Loin pain	1.9%	6/314
Splenomegaly	69.7%	255/366^#^
Thrombocytopenia	48.8%	127/260^&^
Anemia	31.4%	69/220^^^
**Clinical treatment and prognosis**		
Open splenectomy	81.0%	325/401
Laparoscopic splenectomy	19.0%	76/401
Diagnosis by percutaneous splenic biopsy	83.3%	10/12
No recurrence or metastasis	91.4%	159/174
Recurrence or metastasis	0.57%	1/174
Death	9.2%	16/174

^*^Total number of patients with symptomatic description.

^$^Upper abdominal discomfort included pain, distension, and discomfort.

^#^Total number of patients with splenomegaly.

^&^Total number of patients with thrombocytopenia.

^^^Total number of patients with anemia.

Furthermore, 174 patients had a clear prognosis (**Table 2**). A total of 159 (91.4%) patients survived without recurrence or metastasis after undergoing splenectomy. However, three patients developed recurrence or metastasis (11, 15, 16). In fact, one patient developed tumor recurrence in the accessory spleen 7 years after undergoing splenectomy, which showed no significant change during the 6-month follow-up (16). According to our statistics, four patients developed LCA both in the spleen and accessory spleen (16, 43, 89, 90). Therefore, it was quite reasonable to find a recurrence of LCA on the unresected accessory spleen. Strictly speaking, this should not be considered as a true relapse or metastasis, and we would rather think that a new LCA has formed in the accessory spleen. In the second patient, the co-occurrence of LCA along with LCHE was detected during splenectomy 8 years ago; the patient unfortunately died of abdominal multiple recurrence and metastasis (11). However, we think that it was the LCHE and not the LCA that recurred and metastasized. Actually, only the third case could be narrowly regarded as recurrence and metastasis, which presented as hepatomegaly with multiple metastatic tumors after splenectomy 10 years and was confirmed by open liver biopsy (15). Even so, the possibility of atypical LCHE or LCAS could not be ruled out.

Sixteen patients died during the follow-up period (1, 11, 12, 15, 39, 53, 58, 59, 91, 92). Among them, eight patients died from concurrent primary malignancies, namely, 2 gastric cancers, 2 malignant lymphomas, 1 multiple myeloma, 1 colon cancer, 1 pancreatic cancer, and 1 ovarian cancer. Four died of postoperative complications. Two patients died at 1.5 and 6 years, respectively, after splenectomy due to unknown causes. The other two patients who died were due to the recurrence and metastasis of the tumor to the liver. In fact, one of them died due to the recurrence and metastasis of LCHE (11), while the other died of hemorrhagic cerebral infarction 21 months after the diagnosis of intrahepatic metastatic LCA (15). Obviously, these causes of death had no direct correlation with LCA.

### Imaging Features of the LCA Patients

**Figure 3:**
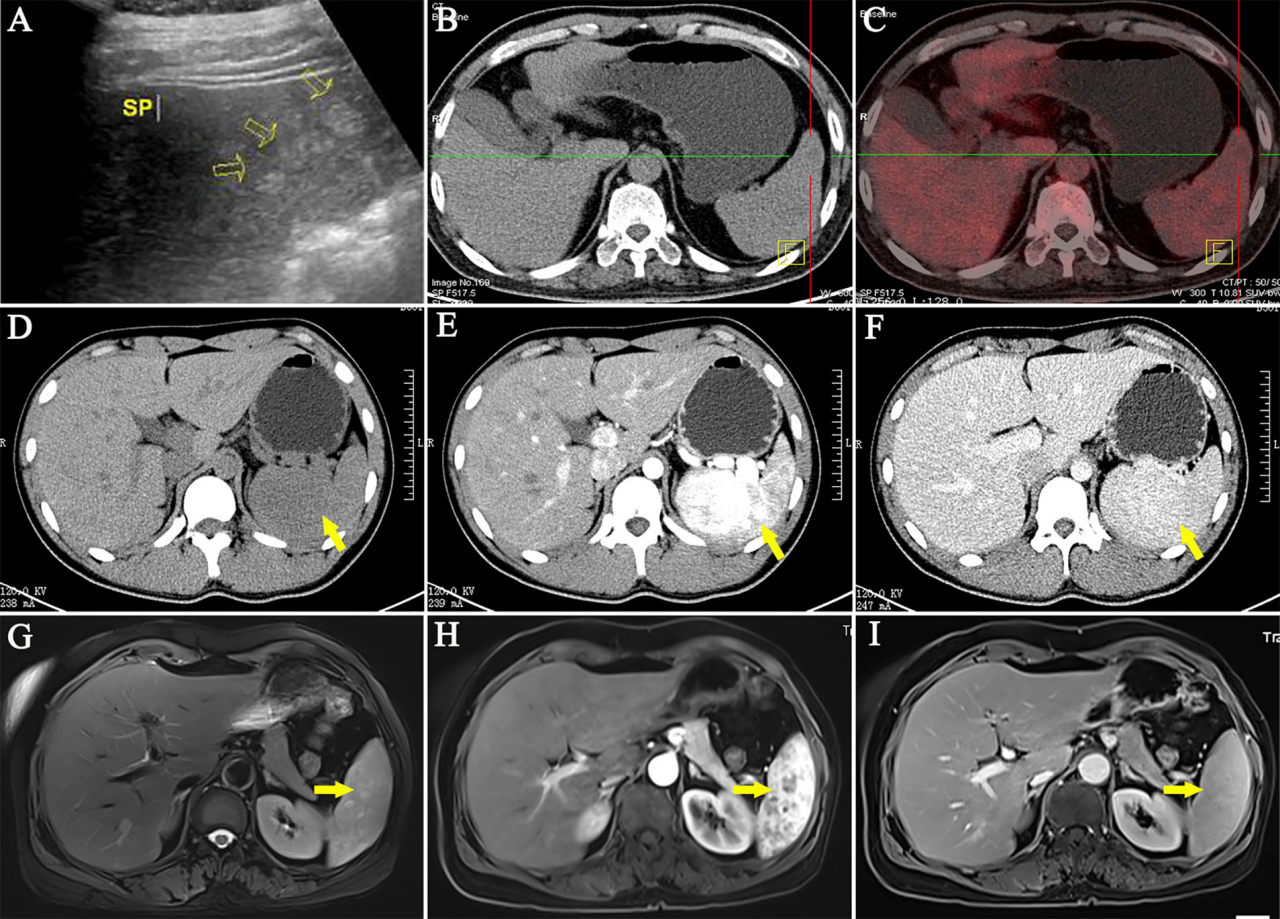
Representative imaging of LCA. **(A)** Ultrasound imaging often showed hyperechoic lesions. **(B, C)** The PET/CT showed no abnormal uptake of 18F-FDG. **(D)** On nonenhanced CT, LCA often manifested as single or multiple hypodense nodules. **(E, F)** On enhanced CT, LCA characterized by delayed reinforcement. **(G)** In T2WI, the splenic lesions mostly manifested as a high signal intensity. **(H, I)** Similar to contrast-enhanced CT, Enhanced MRI showed a delayed enhancement.

First, the ultrasound (US) features of 92 LCA patients are summarized in **Table 3**. LCA manifested as hyperechoic (**Figure 3A**), hypoechoic, isoechoic, and heterogeneous echo in 43.5, 35.9, 9.8, and 10.9% of the patients, respectively. On the color Doppler imaging of 25 LCA patients, the color flow signals were 76% of hypovascular and 24% of hypervascular. There were relatively few data on the contrast-enhanced ultrasound (CEUS) findings. All 8 LCA patients who underwent CEUS showed enhancement in arterial phase. Of the 7 patients with portal phase description, 6 patients had enhanced features in CEUS. Only 3 patients had a description of delayed phase, and 2 patients presented enhancement. Second, 214 LCA patients underwent computed tomography (CT). On nonenhanced CT, 84.5% showed hypodense (**Figures 3B, D** **)**, 13.9% isodense, and 1.6% showed hyperdense lesions. On enhanced CT, 125 LCA patients underwent arterial phase imaging, 58.4% presented enhancement. Of the 153 patients with portal phase imaging, 77.1% showed enhancement. Of the 96 patients with delayed phase imaging, 94.8% characterized by delayed enhancement (**Figures 3E, F** **)**. Third, the magnetic resonance imaging (MRI) features of 66 LCA patients were summarized. On unenhanced T1-weighted images (T1WI), the lesions were depicted as hypointense masses in 75.9% of 58 patients. Moreover, 17.2 and 6.9% of patients showed isosignal intensity and high signal intensity, respectively. On unenhanced T2-weighted images (T2WI), the splenic lesions mostly manifested as a high signal intensity (**Figure 3G**) in 76.6% of 64 patients. However, they also showed hypointense, isointense, and mixed signal intense masses in 18.8, 3.1, and 1.6% of the patients, respectively. Of the 30 patients with DWI description, 90% showed hyperintense lesions, and only 10% showed hypointense lesions. On enhanced MRI, 43 LCA patients underwent arterial phase imaging, and 62.8% presented enhancement. Of the 45 LCA patients with portal phase imaging, 88.9% showed enhancement. Of the 30 patients with delayed phase imaging, 93.3% characterized by delayed enhancement (**Figures 3H, I** **)**.

**Table 3:** Imaging features of the LCA patients.

Imaging features	Value	N. With data reported
**Ultrasound imaging**		**92**
Hyperechoic	43.5%	40/92
Hypoechoic	35.9%	33/92
Isoechoic	9.8%	9/92
Heterogeneous echo	10.9%	10/92
Hypovascular	76%	19/25
Hypervascular	24%	6/25
Enhancement in arterial phase of CEUS^*^	100%	8
Enhancement in portal venous phase of CEUS	85.7%	6/7
Enhancement in delayed phase of CEUS	66.7%	2/3
**Computed tomography imaging**		**214**
Hypodense in CT scan	84.5%	158/187
Isodensein CT scan	13.9%	26/187
Hyperdensein CT scan	1.6%	3/187
Enhancement in arterial phase	58.4%	73/125
Enhancement in portal venous phase	77.1%	118/153
Enhancement in delayed phase	94.8%	91/96
**Magnetic resonance imaging**		**66**
T1 hyperintense	6.9%	4/58
T1 hypointense	75.9%	44/58
T1 isointensity	17.2%	10/58
T2 hyperintense	76.6%	49/64
T2 hypointense	18.8%	12/64
T2 isointensity	3.1%	2/64
T2 mixed signal intensity	1.6%	1/64
DWI hyperintense	90%	27/30
DWI hypointense	10%	3/30
Enhancement in arterial phase	62.8%	27/43
Enhancement in portal venous phase	88.9%	40/45
Enhancement in delayed phase	93.3%	28/30
**PET/CT and SPECT imaging**		**19**
FDG uptake	22.2%	2/9
No abnormal FDG uptake	77.8%	7/9
High uptake of radionuclides	30%	3/10
No abnormal radionuclides uptake	70%	7/10

^*^CEUS, Contrast-enhance Ultrasound.

Bold letters represent different types of imaging examination and are a summary of the total number of published cases with imaging findings.

In addition, 19 LCA patients underwent radionuclide imaging (**Table 3**). In nine patients who underwent positron emission tomography/CT examination, 7 who developed the splenic lesions had no abnormal uptake (11, 32, 33, 35, 36, 65, 93) (**Figure 3C**). However, 2 patients showed high uptake of 18F-fluorodeoxyglucose (11, 94). The other 10 patients underwent single-photon emission computed tomography, of whom 6 showed 99 mTc uptake (76, 80, 95–98), 2 showed Ga uptake (47, 89), and 2 had undescribed radionuclides (28, 99). Based on these results, only 30% of the patients showed a high uptake.

### Characteristics of Spleen and LCA

The size and weight of the spleen slightly to moderately increased (**Table 4**). In the splenomegaly group, the median splenic volume and weight were 1,140 cm^3^ and 575 g, with the maximum volume and weight being 5,040 cm^3^ and 4,018 g, respectively. Meanwhile, the median splenic volume and weight were only 389 cm^3^ and 187 g, respectively, in the normal-sized spleens. Furthermore, LCA could appear as multifocal lesions or a solitary nodule with a solitary-to-multiple ratio of 0.31 in 349 patients. The average diameter of solitary LCA was 6.4 ± 4.0 cm, with the maximum diameter being 21 cm (31). The average diameter of solitary LCA was only 3.3 ± 2.6 cm in the multiple LCA group, with the maximum diameter being 18 cm (1).

**Table 4:** Characteristics of spleen and LCA.

Characteristics	Value	N. With data reported
Splenomegaly/Normal	2.3	255/111
Volume of splenomegaly (range, median, IQR)	203–5,040 cm^3^, 1,140 cm^3^, 1,119 cm^3^	75/105
Volume of normal spleen (range, median, IQR)	98–840 cm^3^, 389 cm^3^, 239 cm^3^	30/105
Weight of splenomegaly (range, median, IQR)	200–4,018 g, 575 g, 590 g	71/93
Weight of normal spleen (range, median, IQR)	120–300 g, 187 g, 64 g	22/93
Solitary/Multiple	0.31	83/266
The diameter of solitary LCA (range, mean, SD)	0.2–21 cm, 6.4 cm, 4 cm	66^*^
The diameter of multiple LCA (range, mean, SD)	0.2–18 cm, 3.3 cm, 2.5 cm	142^*^
Period for LCA formation (range, median, IQR)	0.87–48 m, 15 (14.3) m	10^#^
Follow-up period with untreated LCA(range, median, IQR)	0.5–108 m, 24 (56.5) m	11^&^

^*^The maximum tumor diameter.

^#^Detailed description of the period of LCA formation.

^&^Follow-up period of untreated LCA.

A total of 18 patients described the natural history of LCA. Detailed description of the period of LCA formation was provided for ten patients (19, 26, 38, 42, 45, 65, 70, 76, 100, 101). The median time for LCA formation was 15 m, with the shortest and longest being 26 d and 4 y, respectively. Eleven patients with untreated LCA survived during follow-up, which had no obvious symptoms of discomfort (16, 19, 43, 65, 71, 102–107). The median of follow-up time of patients with untreated LCA was 24 m, with the shortest and longest being 0.5 m and 9 y. Although significant enlargement of untreated LCA was observed in three patients, no metastasis occurred. Among them (105–107), two patients developed an increase of the tumor size in the second year, from 0.5 to 3.4 cm and 4.3 cm respectively, and one showed an increase in the tumor size of 9 cm in the ninth year.

### Summary of Histopathology and Immunohistochemical Characteristics of LCA

**Figure 4:**
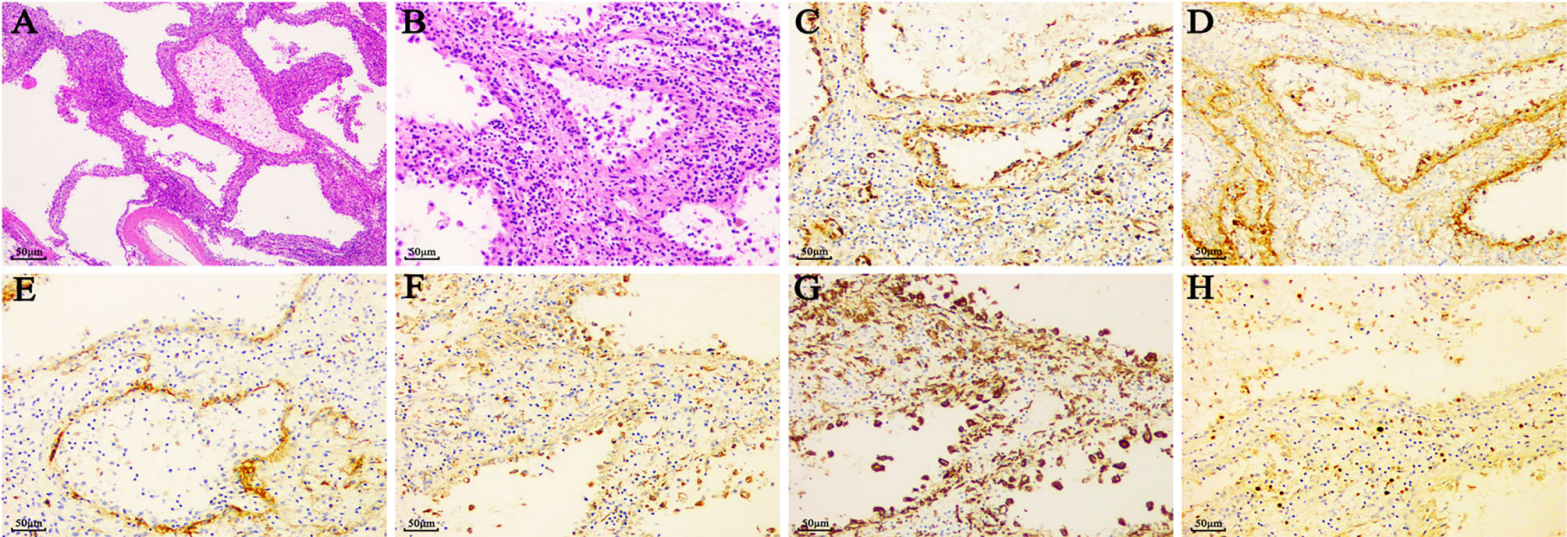
The typical pathological features of LCA. **(A)** (×40) & **(B)** (×200). HE staining of LCA showed sinus like anastomosing channels with an irregular lumen, which was lined with tall, plump endothelial cells. Of the vascular markers, LCA reacted positively with **(C)** CD31, **(D)** factor VIII, and **(E)** CD34. Of the histiocytic markers, LCA typically expressed with **(F)** CD68 and **(G)** CD163. **(H)** A positivity rates of Ki67 was approximately 5%.

Histologically, LCA showed sinus like anastomosing channels with an irregular lumen (**Figure 4**). These channels were either papillary or cystic and lined with tall, plump endothelial cells (1, 3). These endothelial cells exhibited hemophagocytosis and lacked features of nuclear atypia or mitotic activity, which was an important basis to distinguish them from other malignant tumor cells. The electron microscopic examination of LCA showed polygonal tumor cells surrounded by blood vessels (108). Notably, the vessels were only made up of a lining of medium electron density homogenous basilar membrane, which lacked smooth muscles. The tumor cells of LCA had an abundant cytoplasm, namely, mitochondria, a rough endoplasmic reticulum, and lysosomes. Some of them showed intermediate filaments and lipofuscin bodies.

The immunohistochemical staining for littoral cells usually reveals a dual differentiation pattern for both endothelial and histiocytic markers (109) (**Figure 4**, **Table 5**). Of the histiocytic markers, CD163, CD68, lysozyme, CD4, and CD11c showed very similar staining pattern with positivity rates of 100, 99.7, 99.1, 94.7, and 87.5%, respectively. However, factor XIIIa did not react in any cells. Of the vascular markers, all of them reacted positively with CD31, BMA120, UEA-1, FLI1, LYVE1, VEGFR2, claudin-5, and LMO2. A very similar staining pattern was observed with factor VIII and ERG, with positivity rates of 98.4 and 97.2%, respectively. As a tumor suppressor gene, Wilms tumor-1 (WT-1) was involved in development of tissues from the inner layer of intermediate mesoderm and played a role in regulating angiogenesis and proliferation of vascular smooth muscle cells (110). However, all 33 LCA patients showed no reactivity with Wilms tumor-1 (WT-1) (11, 12). Only 54.3% in the 232 LCA patients stained positively for CD34.

**Table 5:** Summary of immunohistochemical characteristics of LCA.

Designation	Specificity	Value	N. With data reported
	**Histiocytic markers**		
CD163	Histiocytes	100%	63/63
CD68	Histiocytes	99.7%	302/303
Lysozyme	Macrophages/Histiocytes, etc.	99.1%	113/114
CD4	Monocyte/macrophage cells/T lymphocyte	94.7%	18/19
CD11c	Dendritic cells/macrophages, etc.	87.5%	14/16
Factor XIII	Macrophages/Histiocytes	0	0/17
	**Endothelial markers**		
CD31	Endothelial cells	100%	276/276
BMA120	Endothelial cells	100%	18/18
UEA-1	Lectin: endothelial cells	100%	17/17
FLI1	Endothelial cells, etc.	100%	17/17
LYVE1	Sinusoidal endothelium, etc.	100%	13/13
VEGFR2	Endothelial cells, etc.	100%	17/17
Claudin-5	Endothelial cells	100%	17/17
LMO2	Endothelial cells	100%	17/17
Factor VIII	Endothelial cells	98.4%	239/243
ERG	Endothelial cells	97.2%	35/36
CD34	Endothelial cells	54.3%	127/234
WT1	A tumor suppressor gene	0	0/33
	**Other indicators with identification**		
CD8	T lymphocytes	11.7%	11/94
CD21	Dendritic cells	57.1%	40/70
S100	Neural structures	38.8%	19/49
D2-40	Lymphatic endothelial cells/mesothelial cells	0	0/24
CK	Epithelium	0	0/37
EMA	Epithelium	7.1%	1/14
Vim	Intermediate filament	65.3%	47/72
Ki67 (range, median, IQR)	Proliferative antigen	80.6% (0–23%, 3%, 4.1%)	50/62^*^

^*^Fifty patients described the positivity rates of ki67, with the highest 23% and the median 3%. Twelve patients reacted positive with ki67, but positivity rates were not described.

CD8 is typically associated with cytotoxic T cells. Its expression by littoral cells highlights the architectural framework of the red pulp in a dendritic pattern (109). Approximately 11.7% of 93 LCA patients stained positively for CD8 (108, 111). Moreover, 57.1% in the 69 patients stained positively for CD21, which is a type I transmembrane protein found on B cells, follicular dendritic cells, pharyngeal and cervical epithelial cells. In addition, S100 is a specific protein used as a marker of neurogenic tumors in pathological diagnosis. In 19 of 49 patients, the tumor cells of LCA exhibited positivity for S100 protein.

Of the lymphatic endothelial markers, D2-40 showed no reactivity in the cells of LCA (108). Similar staining pattern was also observed with epithelium marker CK. Only one of the 14 patients exhibited positivity for epithelium marker EMA (29). As a marker of mesenchymal tissue, the expression of vim indicates that the tissue originates from the mesenchymal rather than the epithelium. Approximately 65.3% of 72 LCA patients stained positively for vim. Notably, 50 patients stained positively for Ki67 with a median rate of 3%, with the minimum and maximum being 0 (112) and 23% (35), respectively. Finally, we proposed a new immunohistochemical phenotype summarized as CD31^+^/ERG^+^/FVIII antigen^+^/CD68^+^/CD163^+^/lysozyme^+^/CD8^−^/WT1^−^ to enhance the differentiation from other primary or secondary splenic tumors.

### A New Mode of Diagnosis and Treatment for LCA

**Figure 5:**
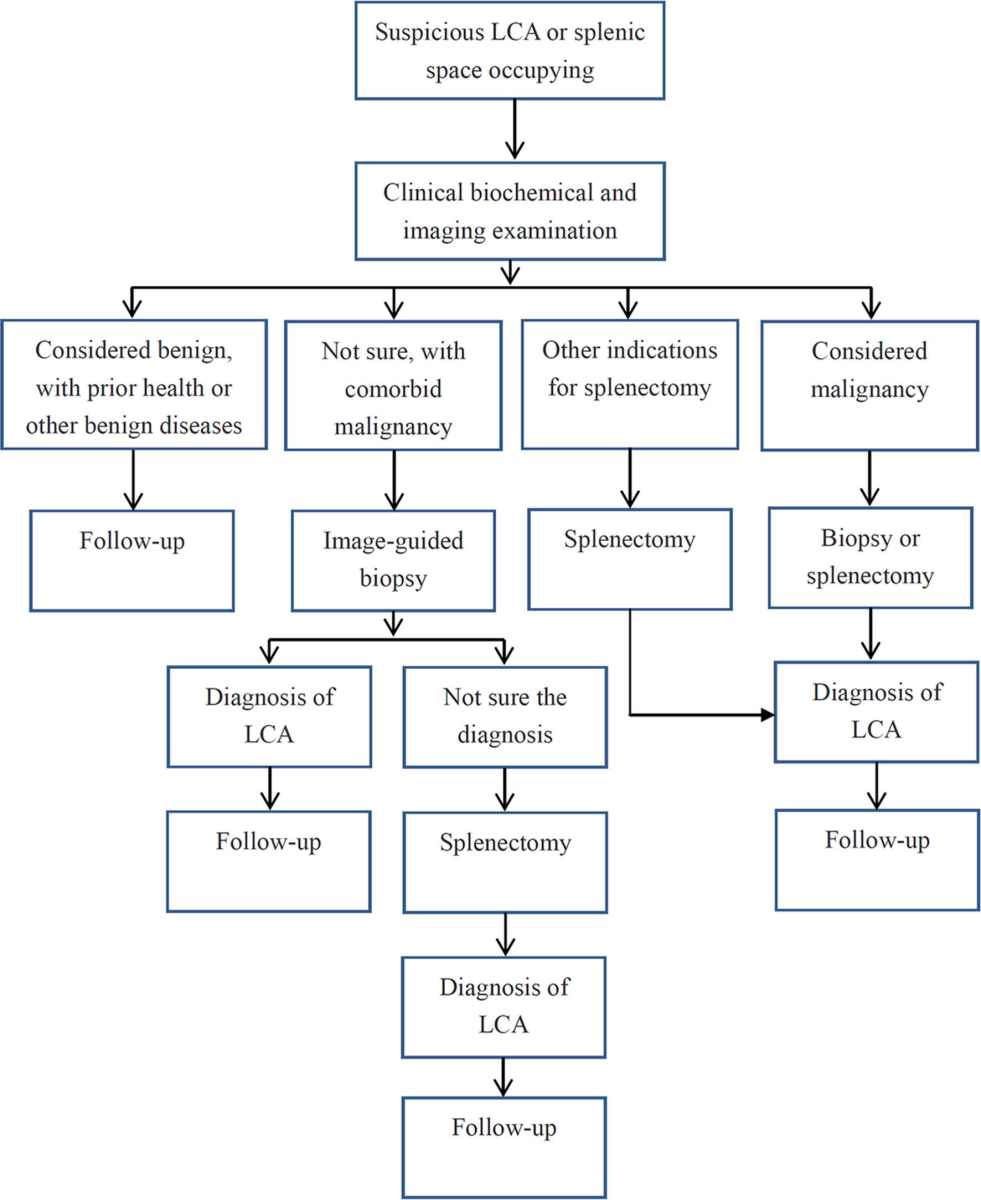
A process of diagnosis and treatment for LCA.

Based on the objective evidence, we herein proposed a process of diagnosis and treatment for LCA (**Figure 5**). For primary splenic space occupying lesions, non-invasive clinical examinations should be performed initially. If the lesion is considered benign, a regular follow-up for 3 to 6 months is recommended. If the nature of the tumor could not be determined or the possibility of metastasis could not be ruled out due to a history of malignant tumor, an image-guided biopsy is suggested. Then, if the tumor is confirmed as LCA without other indications for splenectomy, regular follow-up is still necessary. If it is considered as a malignant lesion, an image-guided biopsy or a direct splenectomy should be performed. In addition, for splenic tumor patients with abdominal discomfort, pathological uncertainty, or continuous tumor enlargement, splenectomy is the preferred treatment. Basically, in order to improve the diagnosis and treatment of this rare disease, regular follow-up is recommended for all patients with definite LCA.

## Discussion

To the best of our knowledge, this was the first systematic review and analysis of all observational studies to describe the clinical landscape of LCA. Due to the low incidence of LCA, studies on LCA published in English and Chinese were collected to summarize the epidemiological characteristics. A total of 435 participants were diagnosed with LCA with a male-to-female ratio of 0.90 (192/213), indicating no gender predilection. The age of LCA onset showed a normal distribution with the oldest and youngest being 86 y (11) and 26 d (19), respectively. Although most of the patients were aged 40–60 years, the incidence of LCA in children was not uncommon (5.7%) (1, 19–30). With regard to the natural history of LCA, 11 patients had an untreated LCA. Among them, LCA was significantly enlarged in three patients but metastasis did not occur. Besides, four patients had a relatively unusual case. One patient had chronic renal failure and underwent dialysis for 1 year, while the other three had malignant tumors. However, no significant changes were observed in the LCA during the follow-up period. Moreover, the natural formation of LCA was documented in 10 patients, with the shortest period being 26 days and the longest period being 4 years. Of the ten patients, four had a previous malignancy, while three had a coexisting immune dysfunction. Therefore, LCA was more like an intra-splenic manifestation of abnormal body function, which could remain stable for a long time or gradually grow in size.

Actually, the etiology and pathogenesis of LCA remains unknown. The available evidences suggested that it was closely related to the occurrence of malignancies (14, 43). The most common malignancies included epithelial, mesenchymal, and hematological malignancies, such as lymphoma, colorectal adenocarcinoma, pancreatic carcinoma, renal adenocarcinoma, malignant melanoma, gastric leiomyosarcoma, and non-small cell carcinoma of the lungs. In a retrospective case study by Peckova, up to 60% of the cases were found to correlate with various visceral malignancies (11). On the contrary, the study by Falk showed an entirely different result, with only 2 of the 17 cases of LCA were associated with malignancy (12%) (1). Based on the results of the systematic analysis, we obtained a similar conclusion and a comorbidity rate of 13.8% (60/435).

In addition, a number of LCA patients showed an association with diseases with presumed immune origin or systemic disease known to cause immune disturbances such as idiopathic thrombocytopenia, ulcerous colitis, Crohn’s disease, autoimmune thrombocytopenia (Evan’s syndrome), Gaucher disease, Epstein syndrome, lymphocytic colitis, myelodysplastic syndrome, chronic glomerulonephritis, aplastic anemia, pulmonary sarcoidosis, post-renal transplantation, hepatitis B and C, psoriasis, chemotherapy, cytostatic treatment, systemic lupus erythematosus, and so on (11, 14). Some studies also speculated that infection and splenic hemodynamic disorder might be related to the formation of LCA (3, 12, 32, 68, 95). Our statistical results showed that a total of 54 LCA patients developed diseases that could lead to immune dysfunction with a comorbidity rate of 12.2% (54/435). Nine of these patients developed both malignant tumors and immune dysfunctions prior to the formation of LCA. It should be emphasized that almost all the malignancies occurred prior to the formation of LCA, and also those immune disorders, indicating that LCA was most likely a response to an internal dysfunction, rather than an initial cause of a body disorder. In summary, immune dysregulation caused by congenital or acquired malignancies, drugs, etc., probably played a vital role in the pathogenesis of LCA.

In terms of clinical characteristics, there is no existing unified and accurate reference index for the diagnosis and treatment of LCA. According to our statistics, patients with LCA were usually asymptomatic (49.7%) or presented with epigastric discomfort (39.2%). Most patients developed splenomegaly (69.7%). About half of them had thrombocytopenia (48.8%), and one-third of them presented with varying degrees of anemia (31.4%). Many scholars were even confused with the morphological features of LCA (82, 113). Most of them only knew that LCA often manifested as multiple lesions. In fact, solitary LCA was not uncommon with a solitary-to-multiple ratio of 0.31 (83/266). In terms of size, LCA ranged from small foci that are unremarkable in the splenic parenchyma to large lesions that are almost completely replacing the splenic parenchyma, with a known maximum diameter of 21 cm (31). Furthermore, a novel panel of immunohistochemical staining for LCA was recommended as CD31^+^/ERG^+^/FVIII antigen^+^/CD68^+^/CD163^+^/lysozyme^+^/CD8^−^/WT1^−^ by summing up the previous pathological findings, which would help distinguish from other primary or secondary splenic tumors.

Imaging findings of LCA correlated well with the gross pathological features. However, US, CT, MRI, or radionuclide imaging did not provide specific findings. They could appear as diverse echoes on ultrasound, and also different densities and signals on CT and MRI with a delayed enhancement generally. Increased ADC values and delayed contrast enhancement on dynamic enhanced T1WI within the LCA lesions suggested a vascular etiology and narrowed the differential diagnosis of multifocal splenic lesions (25, 101). Occasionally, low signal areas were seen on both T1WI and T2WI within the LCA tumors, and remained hypointense after injection of gadolinium (26), which could be due to the significant amounts of hemosiderin in the lesions that caused the formation of a magnetic susceptibility artifact. Notably, most LCA lesions had no abnormal uptake of radionuclide. The overall imaging findings of LCA were consistent with the characteristics of a benign disease.

Finally, the prognosis of LCA has always been the focus of controversy (1, 15, 16, 114). Almost all scholars reported the malignant potential of LCA and the possibility of recurrence, metastasis, or developing malignant tumors. Contrary to the past view, the major findings of this study supported LCA is a benign primary splenic vascular neoplasm without the risk of recurrence or metastasis, which might be a response to an internal dysfunction, rather than an initial cause of other malignancies. According to our statistics, 91.4% (159/174) of the patients survived after undergoing splenectomy without developing adverse events. Meanwhile, 9.2% (16/174) of the patients died during the follow-up period. However, when the causes of death were analyzed, almost all of them were not related to LCA itself. Although three patients experienced recurrence or metastasis, only one could be narrowly regarded as having a real recurrence or metastasis, and the possibility of atypical LCHE or LCAS could not be ruled out. In fact, a similar metastasizing splenic LCHE has been reported by Fernandez, which showed a typical morphology and immune-phenotypes of LCA with a completely bland histological appearance (114). Based on the summary of imaging and pathological characteristics, LCA was more in line with a benign lesion. Postoperative intrahepatic or distant organ metastasis of LCA should be considered as an atypical LCHE or LCAS. Thus, none of the patients with real LCA developed recurrence or metastasis.

However, several limitations should be acknowledged. First, LCA is a rare disease that has originated in the spleen. All published studies had small sample size (<27 patients) (10). The quality of these studies was generally low. Therefore, the recommendations were mainly based on the results of observational studies. Second, the search strategy only included studies indexed in PubMed, Embase, WanFang, and CNKI, published between January 1991 and May 2021 and written in English or Chinese. Third, although a low rate of metastasis was concluded based on the results of observational studies on LCA, we were unable to assess the contribution of splenectomy to this incidence.

In conclusion, this study suggested that LCA was a benign primary splenic vascular neoplasm, which was more like an intra-splenic manifestation of abnormal body function. Accumulated evidence indicated that if LCA could be diagnosed preoperatively, further splenectomy might not be necessary, as it can increase the risk of postoperative complications and is associated with a huge economic cost; only a close follow-up is necessary. For LCA patients with abdominal discomfort, pathological uncertainty or continuous tumor enlargement, splenectomy remains the preferred treatment.

## Data Availability Statement

The original contributions presented in the study are included in the article/**Supplementary Material**. Further inquiries can be directed to the corresponding author.

## Author Contributions

WW and YS designed the study. WW, RZ, and RL collected clinical data. WW, GQ, XZ, and YZ wrote the manuscript. All authors listed have made a substantial, direct, and intellectual contribution to the work and approved it for publication.

## Funding

The study was supported by the National Natural Science Foundation of China (81900558) and the Henan Medical Science and Technique Foundation (SBGJ202003035).

## Conflict of Interest

The authors declare that the research was conducted in the absence of any commercial or financial relationships that could be construed as a potential conflict of interest.

## Publisher’s Note

All claims expressed in this article are solely those of the authors and do not necessarily represent those of their affiliated organizations, or those of the publisher, the editors and the reviewers. Any product that may be evaluated in this article, or claim that may be made by its manufacturer, is not guaranteed or endorsed by the publisher.

## Supplementary Material

Supplementary Data Sheet 1 All the included literatures published in English or Chinese before May 2021.

## References

[1] Falk S Stutte HJ Frizzera G . Littoral Cell Angioma. A Novel Splenic Vascular Lesion Demonstrating Histiocytic Differentiation. Am J Surg Pathol (1991) 15(11):1023–33. doi: 10.1097/00000478-199111000-00001 1928554

[2] Tsang WY Chan JK . Splenic Littoral Cell Angioma, Not Bacillary Angiomatosis. Pathology (1994) 26(3):347. doi: 10.1080/00313029400169831 7991298

[3] Bi C Jiang L Li Z Liu W . Littoral Cell Angioma of Spleen: A Clinicopathologic Study of 17 Cases. Zhonghua Bing Li Xue Za Zhi (2007) 4(36):239–43. doi: 10.3760/j.issn:0529-5807.2007.04.006 17706114

[4] Kaza RK Azar S Al-Hawary MM Francis IR . Primary and Secondary Neoplasms of the Spleen. Cancer Imaging (2010) 10(1):173–82. doi: 10.1102/1470-7330.2010.0026 PMC2943678 20713317

[5] Roldan-Vasquez E Roldan-Vasquez A Jarrin-Estupiñan X Roldan-Crespo J . Case Report: Infrequent Littoral Cell Angioma of the Spleen. Int J Surg Case Rep (2021) 85:106242. doi: 10.1016/j.ijscr.2021.106242 34333257 PMC8346658

[6] Dascalescu CM Wendum D Gorin NC . Littoral-Cell Angioma as a Cause of Splenomegaly. N Engl J Med (2001) 345(10):772–3. doi: 10.1056/NEJM200109063451016 11547761

[7] Levy AD Abbott RM Abbondanzo SL . Littoral Cell Angioma of the Spleen: CT Features With Clinicopathologic Comparison. Radiology (2004) 230(2):485–90. doi: 10.1148/radiol.2302030196 14752189

[8] Akyildiz H Akcan A Soyuer I Ibrahim Karahan O Sozuer E . Littoral Cell Angioma Mimicking Pancreatic Tumor. Surgery (2007) 141(5):690–1. doi: 10.1016/j.surg.2006.04.017 17462471

[9] Gardner JA Devitt K . Incidental Littoral Cell Angioma in Refractory Immune Thrombocytopenic Purpura. Blood (2017) 129(11):1564. doi: 10.1182/blood-2016-10-748772.PMID:28302694 28302694

[10] Cai Y-Q Wang X Ran X Liu X-B Peng B . Laparoscopic Splenectomy for Splenic Littoral Cell Angioma. World J Gastroenterol (2015) 21(21):6660–4. doi: 10.3748/wjg.v21.i21.6660 PMC4458776 26074704

[11] Peckova K Michal M Hadravsky L Suster S Damjanov I Miesbauerova M . Littoral Cell Angioma of the Spleen: A Study of 25 Cases With Confirmation of Frequent Association With Visceral Malignancies. Histopathology (2016) 69(5):762–74. doi: 10.1111/his.13026 27374010

[12] Cao Z Wei J Cen H Yuan X Zhou G Zhao J . 13 Cases of Littoral Cell Angioma in Spleens. Beijing Da Xue Xue Bao Yi Xue Ban (2017) 49:495–500. doi: 10.3969/j.issn.1671-167X.2017.03.020 28628153

[13] Fabiola I Sangiorgio V Arber DA . Vascular Neoplasms and Non-Neoplastic Vascular Lesions of the Spleen. Semin Diagn Pathol (2021) 38(2):154–8. doi: 10.1053/j.semdp.2020.07.001 32674844

[14] Sarandria JJ Escano M Kamangar F Farooqui S Montgomery E Cunningham SC . Massive Splenomegaly Correlates With Malignancy: 180 Cases of Splenic Littoral Cell Tumors in the World Literature. Minerva Chir (2014) 69(4):229–37. 24987971

[15] Takayoshi K Doi G Tsuruta N Yoshihiro T Nio K Tsuchihashi K . Successful Chemotherapeutic Treatment for Metastatic Littoral Cell Angioma: A Case Report. Med (Baltimore) (2018) 97(15):e0378. doi: 10.1097/MD.0000000000010378 PMC5908586 29642193

[16] Venkatanarasimha N Hall S Suresh P Williams MP . Littoral Cell Angioma in a Splenunculus: A Case Report. Br J Radiol (2011) 84(997):e11–3. doi: 10.1259/bjr/60430925 PMC3473802 21172957

[17] Di Sabatino A Carsetti R Corazza GR . Post-Splenectomy and Hyposplenic States. Lancet (2011) 378(9785):86–97. doi: 10.1016/S0140-6736(10)61493-6 21474172

[18] Kristinsson SY Gridley G Hoover RN Check D Landgren O . Long-Term Risks After Splenectomy Among 8149 Cancer-Free American Veterans: A Cohort Study With Up to 27 Years Follow-Up. Haematologica (2014) 99(2):392–8. doi: 10.3324/haematol.2013.092460 PMC3912973 24056815

[19] Gakenheimer-Smith L Mohlman J VandenHeuvel K Jackson WD Thomsen W Stevenson A . A Novel Presentation of Littoral Cell Angioma and Lymphatic Malformations in a Neonate. Pediatrics (2018) 141(Suppl 5):S520–5. doi: 10.1542/peds.2017-2782 29610184

[20] Jasani M Shah A Shah A . Littoral Cell Angioma: A Rare Cause of Pediatric Thrombocytopenia. J Indian Assoc Pediatr Surg (2018) 23(3):156–7. doi: 10.4103/jiaps.JIAPS_214_17 PMC6042161 30050266

[21] Anbardar MH Kumar PV Forootan HR . Littoral Cell Angioma of the Spleen: Cytological Findings and Review of the Literature. J Cytol (2017) 34(2):121–4. doi: 10.4103/JOC.JOC_118_15 PMC5398021 28469325

[22] Bedir R Sehitoǧlu I Calapoǧlu AS Yurdakul C . A Rare Case of Splenic Littoral Cell Angioma in a Child. J Lab Physicians (2014) 6(2):117–20. doi: 10.4103/0974-2727.141511 PMC4196359 25328338

[23] Matuszczak E Reszec J Dębek W Hermanowicz A Chyczewski L . Is Littoral Cell Angioma of the Spleen as Rare as Previously Believed in the Pediatric Population? Folia Histochem Cytobiol (2012) 50(3):480–5. doi: 10.5603/19761 23042283

[24] Forest F Duband S Clemenson A Peoc’h M . Traumatic Subcapsular Splenic Hematoma Revealing Littoral Cell Angioma and Gaucher’s Disease. Ann Hematol (2010) 89(10):1061–2. doi: 10.1007/s00277-010-0909-1 20155266

[25] Ertan G Tekes A Mitchell S Keefer J Huisman TAGM . Pediatric Littoral Cell Angioma of the Spleen: Multimodality Imaging Including Diffusion-Weighted Imaging. Pediatr Radiol (2009) 39(10):1105–9. doi: 10.1007/s00247-009-1339-x 19597808

[26] Oliver-Goldaracena JM Blanco A Miralles M Martin-Gonzalez MA . Littoral Cell Angioma of the Spleen: US and MR Imaging Findings. Abdom Imaging (1998) 23(6):636–9. doi: 10.1007/s002619900420 9922201

[27] Mac New HG Fowler CL . Partial Splenectomy for Littoral Cell Angioma. J Pediatr Surg (2008) 43(12):2288–90. doi: 10.1016/j.jpedsurg.2008.07.031 19040956

[28] Antón-Pacheco J Ayuso RM Cano I Martinez MA Cuadros J Berchi FJ . Splenic Littoral Cell Angioma in an Infant. J Pediatr Surg (2000) 35(3):508–9. doi: 10.1016/s0022-3468(00)90225-2 10726700

[29] Wang C Tian Y Cai W Li N . Littoral Cell Angioma of Spleen Complicated With Hemorrhage – 2019 Film Reading Window (9). Anhui Yixue (2019) 40(9):1076–7. doi: 10.3969/j.issn.1000-0399.2019.09.036

[30] Li Y Wang X Cai Y Peng B . Laparoscopic Central Splenectomy for Littoral Cell Angioma. J Gastrointest Surg (2021) 25(2):576–7. doi: 10.1007/s11605-020-04829-7 33078321

[31] Truong V Finch R Martin B Buzacott K Singh M Patel B . Littoral Cell Angioma of Spleen. ANZ J Surg (2019) 89(4):E158–9. doi: 10.1111/ans.14193 29024432

[32] Fotis K Christina L Ioannis P Angelos K Dionysios L Evangelos K . The Sequence of the Evil: A Case Report of Idiopathic Noncirrhotic Portal Hypertension Associated With Littoral Cell Angioma of the Spleen, 4 Years After the Successful Treatment of a Colon Cancer. J Clin Exp Hepatol (2019) 9(2):273–6. doi: 10.1016/j.jceh.2018.08.004 PMC6477133 31024210

[33] George SA Al Bader I . Incidental Splenic Littoral Cell Angioma Complicating a Case of Rolon Cancer: A Case Report. Gulf J Oncolog (2015) 1(19):14–7. 26499824

[34] Marzetti A Messina F Prando D Verza LA Vacca U Azabdaftari A . Laparoscopic Splenectomy for a Littoral Cell Angioma of the Spleen: Case Report. World J Clin Cases (2015) 3(11):951–5. doi: 10.12998/wjcc.v3.i11.951 PMC4644898 26601099

[35] Sarandria JJ Escano M Kamangar F Farooqui SO Montgomery E Cunningham SC . Littoral Cell Angioma: Gastrointestinal Associations. Gastrointest Cancer Res (2014) 7(2):63–4. PMC4007680 24799975

[36] Melzer N Josef Barth P Müller K-M Foss H-D Krug U Schilling M . Rapidly Progressive B-Cell Dominated Inflammatory Neuropathy and Littoral Cell Angioma of the Spleen Associated With Plasmablastic B-Cell Lymphoma. Leuk Lymphoma (2012) 53(6):1242–4. doi: 10.3109/10428194.2011.640677 22098375

[37] Shah S Wasnik A Pandya A Bude RO . Multimodality Imaging Findings in Image-Guided Biopsy Proven Splenic Littoral Cell Angioma: Series of Three Cases. Abdom Imaging (2011) 36(6):735–8. doi: 10.1007/s00261-011-9697-x 21318377

[38] Pilz JB Sperschneider T Lutz T Loosli B Maurer CA . Littoral Cell Angioma in Main and Accessory Intrapancreatic Spleen Presenting as Splenic Rupture. Am J Surg (2011) 201(2):e15–7. doi: 10.1016/j.amjsurg.2009.11.013 20409532

[39] Priego P Rodríguez Velasco G Griffith PS Fresneda V . Littoral Cell Angioma of the Spleen. Clin Transl Oncol (2008) 10(1):61–3. doi: 10.1007/s12094-008-0155-3 18208795

[40] Bhatt S Huang J Dogra V . Littoral Cell Angioma of the Spleen. AJR Am J Roentgenol (2007) 188(5):1365–6. doi: 10.2214/AJR.06.1157 17449783

[41] Harmon RL Cerruto CA Scheckner A . Littoral Cell Angioma: A Case Report and Review. Curr Surg (2006) 63(5):345–50. doi: 10.1016/j.cursur.2006.06.011 16971207

[42] Mohan V Jones RC Drake AJ 3rd Daly PL Mohamed Shakir KM . Littoral Cell Angioma Presenting as Metastatic Thyroid Carcinoma to the Spleen. Thyroid (2005) 15(2):170–5. doi: 10.1089/thy.2005.15.170 15753678

[43] Floyd JD Kaplan PA Sauter ER Perry MC Doll DC . Patients With Unusual Bladder Malignancies and a Rare Cause of Splenomegaly. Case 3. Littoral Cell Angioma of the Spleen in a Patient With Previous Lymphoma. J Clin Oncol (2005) 23(19):4460–2. doi: 10.1200/JCO.2005.05.066 15994157

[44] Heese J Bocklage T . Specimen Fine-Needle Aspiration Cytology of Littoral Cell Angioma With Histologic and Immunohistochemical Confirmation. Diagn Cytopathol (2000) 22(1):39–44. doi: 10.1002/(sici)1097-0339(200001)22:1<39::aid-dc11>3.0.co;2-q 10613972

[45] Steensma DP Morice WG . Littoral Cell Angioma Associated With Portal Hypertension and Resected Colon Cancer. Acta Haematol (2000) 104(2-3):131–4. doi: 10.1159/000039747 11154990

[46] Bisceglia M Sickel JZ Giangaspero F Gomes V Amini M Michal M . Littoral Cell Angioma of the Spleen: An Additional Report of Four Cases With Emphasis on the Association With Visceral Organ Cancers. Tumori (1998) 84(5):595–9. doi: 10.1177/030089169808400516 9862523

[47] Yano H Imasato M Monden T Okamoto S . Hand-Assisted Laparoscopic Splenectomy for Splenic Vascular Tumors: Report of Two Cases. Surg Laparosc Endosc Percutan Tech (2003) 13(4):286–9. doi: 10.1097/00129689-200308000-00014 12960796

[48] Selove W Picarsic J Swerdlow SH . Langerin Staining Identifies Most Littoral Cell Angiomas But Not Most Other Splenic Angiomatous Lesions. Hum Pathol (2019) 83:43–9. doi: 10.1016/j.humpath.2018.08.012 30130631

[49] Schneider G Uder M Altmeyer K Bonkhoff H Gruber M Kramann B . Littoral Cell Angioma of the Spleen: CT and MR Imaging Appearance. Eur Radiol (2000) 10(9):1395–400. doi: 10.1007/s003300000345 10997426

[50] Chatelain D Bonte H Guillevin L Balladur P Flejou J-F . Small Solitary Littoral Cell Angioma Associated With Splenic Marginal Zone Lymphoma and Villous Lymphocyte Leukaemia in a Patient With Hepatitis C Infection. Histopathology (2002) 41(5):473–5. doi: 10.1046/j.1365-2559.2002.14313.x 12405918

[51] Mokhtari N Hamidian Jahromi A Dela Cruz N Woodward A Do D Thomas-Ogunniyi JO . Littoral Cell Angioma: Review of the Literature and Case Report. J La State Med Soc (2013) 165(6):329–33. 25073259

[52] Lin C-H Yu J-C Shih M-L Peng Y-J Hsieh C-B . Littoral Cell Angioma of the Spleen in a Patient With Hepatocellular Carcinoma. J Formos Med Assoc (2005) 104(4):282–5. 15909068

[53] Dong W Li F Kang Q Li Y Chen Y Ma J . Diagnosis and Treatment of 6 Cases of Splenic Littoral Cell Angioma. Zhejiang Med (2019) 41(15):1653–4. doi: 10.12056/j.issn.1006-2785.2019.41.15.2017-3046

[54] Liu H Liu M Liu Y Xiao W Xu S . Imaging Features of Splenic Littoral Cell Angioma. Chin J Radiol (2013) 47(5):440–3. doi: 10.3760/cma.j.issn.1005-1201.2013.05.012

[55] Deng L Qiu M Zhang S . Littoral Cell Angiomas of the Spleen : Imaging Features With Pathological Comparison. Chin J Med Computed Imaging (2008) 14(5):415–9. doi: 10.3969/j.issn.1006-5741.2008.05.009

[56] Huang G Wang J Liu B Wang H Yu Y . MSCT Imaging Features of the Littoral Cell Angioma (LCA) in Spleen [5-Case Report]. Chin J CT MRI (2014) 4:107–10. doi: 10.3969/j.issn.1672-5131.2014.04.33

[57] He J Shen J Zhou W Zhu Y . Littoral Cell Angiomas of the Spleen: CT and MRI Findings With Pathological Comparison. J Med Imaging (2015) 3:489–91.

[58] Chen J . Splenic Littoral Cell Angioma: A Clinicopathologic Analysis of Three Cases. Zhejiang Pract Med (2007) 12(3):177–9. doi: 10.3969/j.issn.1007-3299.2007.03.013

[59] Hu Y Shi Q . Pathological Analysis of Splenic Littoral Cell Angioma: A Report of 6 Cases. J Chin Oncol (2011) 17(4):311–2.

[60] Li X Xu Q . CT Features of Splenic Littoral Cell Angioma (4 Cases Report and Literature Review). J Pract Radiol (2016) 32(7):1154–5. doi: 10.3969/j.issn.1002-1671.2016.07.046

[61] Zhang S Zhang S Yan X Liu G . Relationship Between CT and MR Features and Pathological Features in Littoral Cell Angioma. Chin J Difficult Complicated Cases (2018) 17(12):1380–3. doi: 10.3969/j.issn.1671-6450.2018.12.019

[62] Jing M Wang A Yang Y Xie G . Splenic Littoral Cell Angioma: A Clinicopathologic Analysis of Two Cases. Pract J Clin Med (2018) 15(1):217–8. doi: 10.3969/j.issn.1672-6170.2018.01.072

[63] Du J Wu B Jin X Zhou H Shi Q Zhou X . Splenic Littoral Cell Angioma: A Clinicopathologic Analysis of Three Cases. Chin J Diagn Pathol (2010) 17(2):107–10. doi: 10.3969/j.issn.1007-8096.2010.02.08

[64] Fang W Wang L Fan Q Jiang Z Wu X . Littoral Cell Angioma of Spleen: A Clinicopathologic Analysis of Two Cases. Chin J Diagn Pathol (2011) 18(1):56–9. doi: 10.3969/j.issn.1007-8096.2011.01.016

[65] Opatrny V Treska V Waloschek T Molacek J . Littoral Cell Angioma of the Spleen: A Case Report. SAGE Open Med Case Rep (2020) 8:2050313X20959874. doi: 10.1177/2050313X20959874 PMC7543100 33088569

[66] Johansson J Björnsson B Ignatova S Sandström P Ekstedt M . Littoral Cell Angioma in a Patient With Crohn’s Disease. Case Rep Gastrointest Med (2015) 2015:474969. doi: 10.1155/2015/474969 25705528 PMC4326338

[67] Nagarajan P Cai G Padda MS Selbst M Kowalski D Proctor DD . Littoral Cell Angioma of the Spleen Diagnosed by Endoscopic Ultrasound-Guided Fine-Needle Aspiration Biopsy. Diagn Cytopathol (2011) 39(5):318–22. doi: 10.1002/dc.21384 21488173

[68] Cordesmeyer S Pützler M Titze U Paulus H Hoffmann MW . Littoral Cell Angioma of the Spleen in a Patient With Previous Pulmonary Sarcoidosis: A TNF-Alpha Related Pathogenesis? World J Surg Oncol (2011) 9:106. doi: 10.1186/1477-7819-9-106 21929754 PMC3187736

[69] Bierenbaum J Alapat DV Godinez C Park AE Frank Zhao X Baer MR . Littoral Cell Angioma: A Correctable Cause of Progressive Pancytopenia in a Patient With Myelodysplastic Syndrome. Leuk Res (2010) 34(4):e117–9. doi: 10.1016/j.leukres.2009.09.030 19853914

[70] Susanne Mühlfeld A Eitner F Perez-Bouza A Knuechel R Heintz B Floege J . Littoral Cell Angioma of the Spleen Mimicking Posttransplantation Lymphoma in a 63-Year-Old Renal Transplant Patient. Am J Kidney Dis (2008) 52(3):e11–4. doi: 10.1053/j.ajkd.2008.01.033 18479795

[71] Suto H Imai H Sato E Ando J Nobukawa B Sugimoto K . Severe Thrombocytopenia Caused by Littoral Cell Angioma. Int J Hematol (2008) 88(3):253–4. doi: 10.1007/s12185-008-0162-8 18766305

[72] Wilsher MJ . Littoral Cell Angioma and Splenic Lipogranulomata in a Renal Dialysis Patient With Chronic Left Loin Pain. Pathology (2006) 38(3):277–9. doi: 10.1080/00313020600699243 16753761

[73] Blansfield JA Goldhahn RT Jr Josloff RK . Littoral Cell Angioma of the Spleen Treated by Laparoscopic Splenectomy. JSLS (2005) 9(2):222–4. PMC3015579 15984716

[74] Kim H-G Park I-S Lee J-I Jeong S Lee J-W Kwon K-S . Littoral Cell Angioma (LCA) Associated With Liver Cirrhosis. Yonsei Med J (2005) 46(1):184–8. doi: 10.3349/ymj.2005.46.1.184 PMC2823050 15744827

[75] Tan YM Chuah KL Wong WK . Littoral Cell Angioma of the Spleen. Ann Acad Med Singap (2004) 33(4):524–6. 15329769

[76] Tholouli E Roulson J-A Byers R Burton I Liu Yin JA . Littoral Cell Angioma of the Spleen in a Patient With Severe Aplastic Anaemia. Haematologica (2003) 88(9):ECR30. 12969823

[77] Gupta MK Levin M Aguilera NS Pastores GM . Littoral Cell Angioma of the Spleen in a Patient With Gaucher Disease. Am J Hematol (2001) 68(1):61–2. doi: 10.1002/ajh.1151 11559940

[78] Sallah S Gonzalez P Maia DM Kelekis N Semelka R . Littoral Cell Angioma in a Patient With Epstein Syndrome. Acta Haematol (1997) 98(2):113–5. doi: 10.1159/000203601 9286309

[79] Goldfeld M Cohen I Loberant N Mugrabi A Katz I Papura S . Littoral Cell Angioma of the Spleen: Appearance on Sonography and CT. J Clin Ultrasound (2002) 30(8):510–3. doi: 10.1002/jcu.10101 12242742

[80] Johnson C Goyal M Kim B Wasdahl D Nazinitsky K . Littoral Cell Angioma. Clin Imaging (2007) 31(1):27–31. doi: 10.1016/j.clinimag.2006.09.021 17189843

[81] Erçin C Gurbuz Y Hacihanefioğlu A Turgut Karakaya A . Multiple Littoral Cell Angioma of the Spleen in a Case of Myelodysplastic Syndrome. Hematology (2005) 10(2):141–4. doi: 10.1080/10245330400026121 16019460

[82] Li M-J Zhou X Cao J-Y Zhu C-Z Zhou S-S Zang Y-J . Laparoscopic Splenectomy for Littoral Cell Angioma of the Spleen: A Case Report. Med (Baltimore) (2019) 98(11):e14825. doi: 10.1097/MD.0000000000014825 PMC6426590 30882665

[83] Cheng S-P Yang T-L Chen B-F Liu C-L . Image of the Month. Littoral Cell Angioma. Arch Surg (2005) 140(11):1127–8. doi: 10.1001/archsurg.140.11.1127-a 16301452

[84] Li G Le M Gao H . Clinicopathologic Features of Littoral Cell Angioma in Spleen. Chin J Diagn Pathol (2011) 18(2):110–2. doi: 10.3969/j.issn

[85] Jiang H Tong J Da J . Littoral Cell Angioma of the Spleen: One Case Report and a Twenty-Year Review of the Literature. Chin J Surg Oncol (2011) 3(6):321–4. doi: 10.3969/j.issn.1674-4136.2011.06.001

[86] Zhang L Chen Y . Clinicopathological Analysis of Splenic Littoral Cell Angioma. Chin J Clin Exp Pathol (2011) 27(1):99–100. doi: 10.3969/j.issn.1001-7399.2011.01.026

[87] Yang D Qiu C Liu D Sun W Zhang M He P . Littoral Cell Angioma of the Spleem: A Case Report. Chin J Diagn Pathol (2008) 15(6):488–9. doi: 10.3969/j.issn.1007-8096.2008.06.016

[88] Yan Z Wu X Zhan H Zhang G Hu S . Laparoscopic Splenectomy for Littoral Cell Angioma of the Spleen:a Report of 3 Cases and Review of the Literature. J Laparoscopic Surg (2017) 22(8):588–91. doi: 10.13499/j.cnki.fqjwkzz.2017.08.588

[89] Chang M-K Sudar S C Gupta R Sawhney H Abdu A Kuo H-Y . Extramedullary Hemopoiesis With Littoral Cell Angioma Involving Main and Accessory Spleens. Ann Hematol (2007) 86(9):695–6. doi: 10.1007/s00277-007-0311-9 17516067

[90] Pillay Y Shokeir MO . Case Report of a Littoral Cell Angioma of the Spleen and Accessory Spleens: A Benign Vascular Tumour. Int J Surg Case Rep (2017) 40:109–12. doi: 10.1016/j.ijscr.2017.09.017 PMC5633815 28965086

[91] Li Q Wang L Zhou X Wang D Li X . Spontaneous Rupture of Splenic Littoral Cell Angioma: A Case Report. J Binzhou Med Coll (2015) 5:395–7.

[92] Luo X Chen G Cao Y . Hemangioma of Spleen Sinus: One Case Report. J Med Imaging (2018) 28(7):1064–8.

[93] Lu J Liang W Zhong D Zhang Y Zhu Z Qin M . Littoral Cell Angioma of the Spleen: Clinical, Pathological Findings and Imaging Features. Chin J Med Imaging (2012) 20(5):368–71. doi: 10.3969/j.issn.1005-5185.2012.05.013

[94] Ji X Yu J Shi X Wang Z Li H Han X . Splenic Littoral Cell Angioma: A Report of a Cases and Literature Review. J Mod Oncol (2018) 26(11):1757–9. doi: 10.3969/j.issn.1672-4992.2018.11.026

[95] Berman E Ikpatt F Wang D Dembner A Zauber NP . Rapid Progression of Littoral Cell Angioma of the Spleen in a Man With Multiple Infections. Rare Tumors (2010) 2(1):e17. doi: 10.4081/rt.2010.e17 21139945 PMC2994494

[96] Tee M Vos P Zetler P Wiseman SM . Incidental Littoral Cell Angioma of the Spleen. World J Surg Oncol (2008) 6:87. doi: 10.1186/1477-7819-6-87 18713469 PMC2527567

[97] Suvajdzić N Cemerikić-Martinović V Saranović D Petrović M Popović M Artiko V . Littoral-Cell Angioma as a Rare Cause of Splenomegaly. Clin Lab Haematol (2006) 28(5):317–20. doi: 10.1111/j.1365-2257.2006.00801.x 16999722

[98] Barshack I Perelman M Many A Goshen E Zwas ST Kopolovic J . Littoral Cell Angioma: A Vascular Tumor Mimicking a Solid Tumor on a Tc-99m-Red Blood Cell Spleen Scan. Isr J Med Sci (1997) 33(10):677–80. 9397143

[99] Yuan X Zhang J . A Case of Spleen Sinus Hemangioma. Chin J Clin Electron Ed (2010) 4(10):2054–5. doi: 10.3969/cma.j.issn.1674-0785.2010.10.075

[100] Ursuleac I Iosif C Bîrlă R Dobrea C Găman AM Arsene D . Littoral Cell Angioma of the Spleen–A Surprising Cause of Anemia. Rom J Morphol Embryol (2013) 54(3 Suppl):885–8. 24322045

[101] Kinoshita LL Yee J Nash SR . Littoral Cell Angioma of the Spleen: Imaging Features. AJR Am J Roentgenol (2000) 174(2):467–9. doi: 10.2214/ajr.174.2.1740467 10658726

[102] Leung VA Tang S Mahe E Patlas MN . Littoral Cell Angioma: Diagnosis by Image-Guided Biopsy. Ann Clin Lab Sci (2012) 42(4):417–21. 23090739

[103] Ramdall RB Alasio TM Cai G Yang GCH . Primary Vascular Neoplasms Unique to the Spleen: Littoral Cell Angioma and Splenic Hamartoma Diagnosis by Fine-Needle Aspiration Biopsy. Diagn Cytopathol (2007) 35(3):137–42. doi: 10.1002/dc.20568 17304535

[104] Lin Y Xu X Jiang X . A Case of Splenic Littoralcellangioma and Literature Review. Hainan Med J (2015) 16:2481–2. doi: 10.3969/j.issn.1003-6350.2015.16.0899

[105] Wang Y-J Li F Cao F Sun J-B Liu J-F Wang Y-H . Littoral Cell Angioma of the Spleen. Asian J Surg (2009) 32(3):167–71. doi: 10.1016/S1015-9584(09)60389-4 19656757

[106] Cao F Wang Y Li F Sun J Liu J Wang Y . A Case of Splenic Littoral Cell Angioma. Chin J Surg (2008) 46(10):727. doi: 10.3321/j.issn:0529-5815.2008.10.026

[107] Li R Cao M Xu J Zhou B Deng M . A Case of Splenic Littoral Cell Angioma. J Gen Surg (2018) 33(11):974–5. doi: 10.3760/cma.j.issn.1007-631X.2018.11.028

[108] Du J Shen Q Yin H Zhou X Wu B . Littoral Cell Angioma of the Spleen: Report of Three Cases and Literature Review. Int J Clin Exp Pathol (2015) 8(7):8516–20. PMC4555755 26339427

[109] Borch WR Aguilera NS Brissette MD O’Malley DP Auerbach A . Practical Applications in Immunohistochemistry: An Immunophenotypic Approach to the Spleen. Arch Pathol Lab Med (2019) 143(9):1093–105. doi: 10.5858/arpa.2018-0211-CP 30917045

[110] O’Malley DP Kim YS Weiss LM . Distinctive Immunohistochemical Staining in Littoral Cell Angioma Using ERG and WT-1. Ann Diagn Pathol (2015) 19(3):143–5. doi: 10.1016/j.anndiagpath.2015.02.007 25792460

[111] Fadar O Hileeto D Mariappan MR . Pathologic Quiz Case: Multiple Splenic Lesions in a Bacteremic Patient. Littoral Cell Angioma of the Spleen. Arch Pathol Lab Med (2004) 128(10):1183–5. doi: 10.1043/1543-2165(2004)128<1183:pqcmsl>2.0.co;2 15387696

[112] Yuhai B Zhiyong S Xingzhi M Xiaolu Y Qiang L Hui C . Two Cases of Littoral Cell Angioma. Chin J Dig Surg (2008) 7(03):232–4.

[113] Lyu S He Q . Huge Littoral Cell Angioma of the Spleen: A Case Report. J Nippon Med Sch (2019) 86(3):179–82. doi: 10.1272/jnms.JNMS.2019_86-307 31292330

[114] Fernandez S Cook GW Arber DA . Metastasizing Splenic Littoral Cell Hemangioendothelioma. Am J Surg Pathol (2006) 30(8):1036–40. doi: 10.1097/00000478-200608000-00016 16861977

