# Supplementary material for: Clinical Landscape of Littoral Cell Angioma in the Spleen Based on a Comprehensive Analysis
Source: Front Oncol. 2022 Feb 8;12:790332. doi: 10.3389/fonc.2022.790332 (PMC8861295; doi:10.3389/fonc.2022.790332)
Supplement: Supplementary Data Sheet 1 — All the included literatures published in English or Chinese before May 2021. [file Table_1.docx]

Supplementary Material

# Supplementary Data

**All the Chinese and English literature included.**

1. Ramael M, Schoeters P, De Pooter K, et al. Multi-focal splenic tumour in a belgian patient and a brief review of the literature on littoral cell angioma. Eur J Case Rep Intern Med, 2020, 7(11):001863. DOI: 10.12890/2020_001863.

2. Kumar A, Kindell D, Desai CS. Littoral cell angioma of the spleen. J Gastrointest Surg, 2021, DOI: 10.1007/s11605-021-04980-9.

3. Vaclav O, Vladislav T, Tomas W, et al. Littoral cell angioma of the spleen: a case report. SAGE Open Med Case Rep, 2020, 8:2050313X20959874. DOI: 10.1177/2050313X20959874

4. Li Y, Wang X, Cai Y, et al. Laparoscopic central splenectomy for littoral cell angioma. J Gastrointest Surg, 2021, 25(2): 576-577. DOI: 10.1007/ s11605-020-04829-7.

5. Karapolat B, Kucuk H. A rare vascular tumor of the spleen: littoral cell angioma. Acta Chir Belg, 2020: 1-3. DOI: 10.1080/00015458.2020.1722928.

6. Truong V, Finch R, Martin B, et al. Littoral cell angioma of spleen. ANZ J Surg, 2019, 89(4): E158-158E159. DOI: 10.1111/ans.14193.

7. Fotis K, Christina L, Ioannis P, et al. The sequence of the evil: a case report of idiopathic noncirrhotic portal hypertension associated with littoral cell angioma of the spleen, 4 years after the successful treatment of a colon cancer. J Clin Exp Hepatol, 2019, 9(2): 273-276. DOI: 10.1016/j.jceh.2018.08.004.

8. Takayoshi K, Doi G, Tsuruta N, et al. Successful chemotherapeutic treatment for metastatic littoral cell angioma: a case report. Medicine (Baltimore), 2018, 97(15):e0378. DOI: 10.1097/MD.0000000000010378.

9. Jasani M, Shah A, Shah A. Littoral cell angioma: a rare cause of pediatric thrombocytopenia. J Indian Assoc Pediatr Surg, 2018, 23(3):156-157. DOI: 10.4103/jiaps.JIAPS_214_17.

10. Gakenheimer-Smith L, Mohlman J, VandenHeuvel K, et al. A novel presentation of littoral cell angioma and lymphatic malformations in a neonate. Pediatrics, 2018, 141(Suppl 5):S520-520S525. DOI: 10.1542/peds.2017-2782.

11. Pillay Y, Shokeir MO. Case report of a littoral cell angioma of the spleen and accessory spleens: a benign vascular tumour. Int J Surg Case Rep, 2017, 40:109-112. DOI: 10.1016/j.ijscr.2017.09.017.

12. Anbardar MH, Kumar PV, Forootan HR. Littoral cell angioma of the spleen: Cytological findings and review of the literature. J Cytol, 2017, 34(2): 121-124. DOI: 10.4103/JOC.JOC_118_15.

13. Gardner JA, Devitt K. Incidental littoral cell angioma in refractory immune thrombocytopenic purpura. Blood, 2017, 129(11): 1564. DOI: 10.1182/ blood-2016-10-748772.

14. Peckova K, Michal M, Hadravsky L, et al. Littoral cell angioma of the spleen: a study of 25 cases with confirmation of frequent association with visceral malignancies. Histopathology, 2016, 69(5): 762-774. DOI: 10.1111/his.13026.

15. George SA, Al Bader I. Incidental splenic littoral cell angioma complicating a case of Rolon cancer: A case report. Gulf J Oncolog, 2015, 1(19): 14-17.

16. Marzetti A, Messina F, Prando D, et al. Laparoscopic splenectomy for a littoral cell angioma of the spleen: case report. World J Clin Cases, 2015, 3(11): 951-955. DOI: 10.12998/wjcc.v3.i11.951.

17. Johansson J, Björnsson B, Ignatova S, et al. Littoral cell angioma in a patient with Crohn's disease. Case Rep Gastrointest Med, 2015, 2015: 474969. DOI: 10.1155/2015/474969.

18. de Ridder GG, Galeotti J, Carney J, et al. Persistent thrombocytopaenia in a young man with splenomegaly, rebound thrombocytosis after splenectomy and subsequent pulmonary embolism: splenic littoral cell angioma and associated events. BMJ Case Rep, 2015, 2015. DOI: 10.1136/bcr-2015-212882.

19. Bailey A, Vos J, Cardinal J. Littoral cell angioma: a case report. World J Clin Cases, 2015, 3(10): 894-899. DOI: 10.12998/wjcc.v3.i10.894.

20. Schlitter MC, Rühl H, Thiesler T, et al. Paraneoplastic disseminated intravascular coagulation caused by splenic littoral cell angioma. Ann Hematol, 2014, 93(10):1795-1796. DOI: 10.1007/s00277-014-2060-x.

21. Bedir R, Sehitoǧlu I, Calapoǧlu AS, et al. A rare case of splenic littoral cell angioma in a child. J Lab Physicians, 2014, 6(2): 117-120. DOI: 10.4103/0974-2727.141511.

22. Sarandria JJ, Escano M, Kamangar F, et al. Littoral cell angioma: gastrointestinal associations. Gastrointest Cancer Res, 2014, 7(2):63-64.

23. Emir S, Sozen S, Yazar MF, et al. Littoral-cell angioma of the spleen. Arch Iran Med, 2013, 16(3): 189-191.

24. Cui XW, Ignee A, De Molo C, et al. Littoral cell angioma of the spleen. Z Gastroenterol, 2013, 51(2): 209-212. DOI: 10.1055/s-0032-1325556.

25. Leung VA, Tang S, Mahe E, et al. Littoral cell angioma: diagnosis by image-guided biopsy. Ann Clin Lab Sci, 2012, 42(4): 417-421.

26. Ursuleac I, Iosif C, Bîrlă R, et al. Littoral cell angioma of the spleen--a surprising cause of anemia. Rom J Morphol Embryol, 2013, 54(3 Suppl): 885-888.

27. Gupta P, Peungjesada S, Foshee S, et al. Littoral cell angioma of spleen: an uncommon presentation of a rare neoplasm. J Clin Imaging Sci, 2012, 2:69. DOI: 10.4103/2156-7514.104302.

28. Melzer N, Barth PJ, Müller KM, et al. Rapidly progressive B-cell dominated inflammatory neuropathy and littoral cell angioma of the spleen associated with plasmablastic B-cell lymphoma. Leuk Lymphoma, 2012, 53(6): 1242-1244. DOI: 10.3109/10428194.2011.640677.

29. Matuszczak E, Reszec J, Dębek W, et al. Is littoral cell angioma of the spleen as rare as previously believed in the pediatric population?. Folia Histochem Cytobiol, 2012, 50(3): 480-485. DOI: 10.5603/19761.

30. Kranzfelder M, Bauer M, Richter T, et al. Littoral cell angioma and angiosarcoma of the spleen: report of two cases in siblings and review of the literature. J Gastrointest Surg, 2012, 16(4): 863-867. DOI: 10.1007/s11605-011-1773-6.

31. Jain M, Badwal S, Rastogi A, et al. Littoral cell angioma of spleen in a patient with obstructive jaundice. Indian J Pathol Microbiol, 2012, 55(1): 109-110. DOI: 10.4103/0377-4929.94876.

32. Shah S, Wasnik A, Pandya A, et al. Multimodality imaging findings in image-guided biopsy proven splenic littoral cell angioma: series of three cases. Abdom Imaging, 2011, 36(6): 735-738. DOI: 10.1007/s00261-011-9697-x.

33. Nagarajan P, Cai G, Padda MS, et al. Littoral cell angioma of the spleen diagnosed by endoscopic ultrasound-guided fine-needle aspiration biopsy. Diagn Cytopathol, 2011, 39(5): 318-322. DOI: 10.1002/dc.21384.

34. Venkatanarasimha N, Hall S, Suresh P, et al. Littoral cell angioma in a splenunculus: a case report. Br J Radiol, 2011, 84(997): e11-13. DOI: 10.1259/bjr/60430925.

35. Benetatos N, Filobbos R, Ammori B. Laparoscopic partial splenectomy for littoral cell angioma. J Surg Case Rep, 2011, 2011(7): 4. DOI: 10.1093/jscr/2011.7.4.

36. Pilz JB, Sperschneider T, Lutz T, et al. Littoral cell angioma in main and accessory intrapancreatic spleen presenting as splenic rupture. Am J Surg, 2011, 201(2):e15-17. DOI: 10.1016/j.amjsurg.2009.11.013.

37. Cordesmeyer S, Pützler M, Titze U, et al. Littoral cell angioma of the spleen in a patient with previous pulmonary sarcoidosis: a TNF-α related pathogenesis?. World J Surg Oncol, 2011, 9: 106. DOI: 10.1186/1477-7819-9-106.

38. Bierenbaum J, Alapat DV, Godinez C, et al. Littoral cell angioma: a correctable cause of progressive pancytopenia in a patient with myelodysplastic syndrome. Leuk Res, 2010, 34(4): e117-119. DOI: 10.1016/j.leukres.2009.09.030.

39. Forest F, Duband S, Clemenson A, et al. Traumatic subcapsular splenic hematoma revealing littoral cell angioma and Gaucher's disease. Ann Hematol, 2010, 89(10): 1061-1062. DOI: 10.1007/s00277-010-0909-1.

40. Berman E, Ikpatt F, Wang D, et al. Rapid progression of littoral cell angioma of the spleen in a man with multiple infections. Rare Tumors, 2010, 2(1): e17. DOI: 10.4081/rt.2010.e17.

41. Colović R, Suvajdzić N, Grubor N, et al. Atypical immunophenotype in a littoral cell angioma. Vojnosanit Pregl, 2009, 66(1): 63-65. DOI: 10.2298/vsp0901063c.

42. Rana N, Ming Z, Hui MS, et al. Case report: littoral cell angioma of spleen. Indian J Radiol Imaging, 2009, 19(3): 210-212. DOI: 10.4103/0971-3026.54886.

43. Ertan G, Tekes A, Mitchell S, et al. Pediatric littoral cell angioma of the spleen: multimodality imaging including diffusion-weighted imaging. Pediatr Radiol, 2009, 39(10): 1105-1109. DOI: 10.1007/s00247-009-1339-x.

44. Chourmouzi D, Psoma E, Drevelegas A. Littoral cell angioma, a rare cause of long standing anaemia: a case report. Cases J, 2009, 2: 9115. DOI: 10.1186/1757-1626-2-9115.

45. Tee M, Vos P, Zetler P, et al. Incidental littoral cell angioma of the spleen. World J Surg Oncol, 2008, 6: 87. DOI: 10.1186/1477-7819-6-87.

46. Priego P, Rodríguez Velasco G, Griffith PS, et al. Littoral cell angioma of the spleen. Clin Transl Oncol, 2008, 10(1): 61-63. DOI: 10.1007/s12094-008-0155-3.

47. Tatli S, Cizginer S, Wieczorek TJ, et al. Solitary littoral cell angioma of the spleen: computed tomography and magnetic resonance imaging features. J Comput Assist Tomogr, 2008, 32(5): 772-775. DOI: 10.1097/RCT.0b013e31815614a2.

48. Mühlfeld AS, Eitner F, Perez-Bouza A, et al. Littoral cell angioma of the spleen mimicking posttransplantation lymphoma in a 63-year-old renal transplant patient. Am J Kidney Dis, 2008, 52(3): e11-14. DOI: 10.1053/j.ajkd.2008.01.033.

49. Suto H, Imai H, Sato E, et al. Severe thrombocytopenia caused by littoral cell angioma. Int J Hematol, 2008, 88(3): 253-254. DOI: 10.1007/s12185-008-0162-8.

50. Bhatt S, Simon R, Dogra VS. Littoral cell angioma: sonographic and color doppler features. J Ultrasound Med, 2007, 26(4): 539-542. DOI: 10.7863/ jum.2007.26.4.539.

51. Ramdall RB, Alasio TM, Cai G, et al. Primary vascular neoplasms unique to the spleen: littoral cell angioma and splenic hamartoma diagnosis by fine-needle aspiration biopsy. Diagn Cytopathol, 2007, 35(3): 137-142. DOI: 10.1002/dc.20568.

52. Cosme A, Tejada A, Bujanda L, et al. Littoral-cell angioma of the spleen: a case report. World J Gastroenterol, 2007, 13(48): 6603-6604. DOI: 10.3748/ wjg.v13.i48.6603.

53. Bhatt S, Huang J, Dogra V. Littoral cell angioma of the spleen. AJR Am J Roentgenol, 2007, 188(5): 1365-1366. DOI: 10.2214/AJR.06.1157.

54. Wilsher MJ. Littoral cell angioma and splenic lipogranulomata in a renal dialysis patient with chronic left loin pain. Pathology, 2006, 38(3): 277-279. DOI: 10.1080/ 00313020600699243.

55. Harmon RL, Cerruto CA, Scheckner A. Littoral cell angioma: a case report and review. Curr Surg, 2006, 63(5): 345-350. DOI: 10.1016/j.cursur.2006.06.011.

56. Mohan V, Jones RC, Drake AJ 3rd, et al. Littoral cell angioma presenting as metastatic thyroid carcinoma to the spleen. Thyroid, 2005, 15(2): 170-175. DOI: 10.1089/thy.2005.15.170.

57. Blansfield JA, Goldhahn RT Jr, Josloff RK. Littoral cell angioma of the spleen treated by laparoscopic splenectomy. JSLS, 2005, 9(2): 222-224.

58. Kim HG, Park IS, Lee JI, et al. Littoral cell angioma (LCA) associated with liver cirrhosis. Yonsei Med J, 2005, 46(1): 184-188. DOI: 10.3349/ymj.2005.46.1.184.

59. Floyd JD, Kaplan PA, Sauter ER, et al. Patients with unusual bladder malignancies and a rare cause of splenomegaly. Case 3. Littoral cell angioma of the spleen in a patient with previous lymphoma. J Clin Oncol, 2005, 23(19): 4460-4462. DOI: 10.1200/JCO.2005.05.066.

60. Tan YM, Chuah KL, Wong WK. Littoral cell angioma of the spleen. Ann Acad Med Singap, 2004, 33(4): 524-526.

61. Fadare O, Hileeto D, Mariappan MR. Pathologic quiz case: multiple splenic lesions in a bacteremic patient. littoral cell angioma of the spleen. Arch Pathol Lab Med, 2004, 128(10): 1183-1185. DOI: 10.5858/2004-128-1183-PQCMSL.

62. Levy AD, Abbott RM, Abbondanzo SL. Littoral cell angioma of the spleen: CT features with clinicopathologic comparison. Radiology, 2004, 230(2): 485-490. DOI: 10.1148/radiol.2302030196.

63. Tholouli E, Roulson JA, Byers R, et al. Littoral cell angioma of the spleen in a patient with severe aplastic anaemia. Haematologica, 2003, 88(11): ECR33.

64. Collins PJ, Ettler H, Amann J, et al. Soft-tissue images. Splenic littoral cell angioma. Can J Surg, 2003, 46(3):204-205.

65. Gupta MK, Levin M, Aguilera NS, et al. Littoral cell angioma of the spleen in a patient with Gaucher disease. Am J Hematol, 2001, 68(1): 61-62. DOI: 10.1002/ajh.1151.

66. Dascalescu CM, Wendum D, Gorin NC. Littoral-cell angioma as a cause of splenomegaly. N Engl J Med, 2001, 345(10): 772-773. DOI: 10.1056/ NEJM200109063451016.

67. Kinoshita LL, Yee J, Nash SR. Littoral cell angioma of the spleen: imaging features. AJR Am J Roentgenol, 2000, 174(2): 467-469. DOI: 10.2214/ ajr.174.2.1740467.

68. Heese J, Bocklage T. Specimen fine-needle aspiration cytology of littoral cell angioma with histologic and immunohistochemical confirmation. Diagn Cytopathol, 2000, 22(1):39-44. DOI: 10.1002/(sici)1097-0339(200001)22:1&lt; 39::aid-dc11&gt; 3.0.co;2-q.

69. Español I, Lerma E, Fumanal V, et al. Littoral cell angioma with severe thrombocytopenia. Ann Hematol, 2000, 79(1): 46-49. DOI: 10.1007/s002770050009.

70. Steensma DP, Morice WG. Littoral cell angioma associated with portal hypertension and resected colon cancer. Acta Haematol, 2000, 104(2-3):131-134. DOI: 10.1159/000039747.

71. Bisceglia M, Sickel JZ, Giangaspero F, et al. Littoral cell angioma of the spleen: an additional report of four cases with emphasis on the association with visceral organ cancers. Tumori, 1998, 84(5): 595-599.

72. Oliver-Goldaracena JM, Blanco A, Miralles M, et al. Littoral cell angioma of the spleen: US and MR imaging findings. Abdom Imaging, 1998, 23(6): 636-639. DOI: 10.1007/s002619900420.

73. Sallah S, Gonzalez P, Maia DM, et al. Littoral cell angioma in a patient with Epstein syndrome. Acta Haematol, 1997, 98(2): 113-115. DOI: 10.1159/000203601.

74. Falk S, Stutte HJ, Frizzera G. Littoral cell angioma. A novel splenic vascular lesion demonstrating histiocytic differentiation. Am J Surg Pathol, 1991, 15(11):1023-1033.

75. Yano H, Imasato M, Monden T, et al. Hand-assisted laparoscopic splenectomy for splenic vascular tumors: report of two cases. Surg Laparosc Endosc Percutan Tech, 2003, 13(4): 286-289. DOI: 10.1097/00129689-200308000-00014.

76. López JI, Del Cura JL, De Larrinoa AF, et al. Role of ultrasound-guided core biopsy in the evaluation of spleen pathology. APMIS, 2006, 114(7-8): 492-499. DOI: 10.1111/j.1600-0463.2006.apm_378.x.

77. Suvajdzić N, Cemerikić-Martinović V, Saranović D, et al. Littoral-cell angioma as a rare cause of splenomegaly. Clin Lab Haematol, 2006, 28(5): 317-320. DOI: 10.1111/j.1365-2257.2006.00801.x.

78. Akyildiz H, Akcan A, Soyuer I, et al. Littoral cell angioma mimicking pancreatic tumor. Surgery, 2007, 141(5): 690-691. DOI: 10.1016/j.surg.2006.04.017.

79. Mac New HG, Fowler CL. Partial splenectomy for littoral cell angioma. J Pediatr Surg, 2008, 43(12): 2288-2290. DOI: 10.1016/j.jpedsurg.2008.07.031.

80. Arber DA, Strickler JG, Chen YY, et al. Splenic vascular tumors: a histologic, immunophenotypic, and virologic study. Am J Surg Pathol, 1997, 21(7): 827-835. DOI: 10.1097/00000478-199707000-00011.

81. Selove W, Picarsic J, Swerdlow SH. Langerin staining identifies most littoral cell angiomas but not most other splenic angiomatous lesions. Hum Pathol, 2019, 83: 43-49. DOI: 10.1016/j.humpath.2018.08.012.

82. O&#39, Malley DP, Kim YS, et al. Distinctive immunohistochemical staining in littoral cell angioma using ERG and WT-1. Ann Diagn Pathol, 2015, 19(3): 143-145. DOI: 10.1016/j.anndiagpath.2015.02.007.

83. Schneider G, Uder M, Altmeyer K, et al. Littoral cell angioma of the spleen: CT and MR imaging appearance. Eur Radiol, 2000, 10(9): 1395-1400. DOI: 10.1007/s003300000345.

84. Antón-Pacheco J, Ayuso RM, Cano I, et al. Splenic littoral cell angioma in an infant. J Pediatr Surg, 2000, 35(3): 508-509. DOI: 10.1016/s0022-3468(00)90225-2.

85. Ziske C, Meybehm M, Sauerbruch T, et al. Littoral cell angioma as a rare cause of splenomegaly. Ann Hematol, 2001, 80(1): 45-48. DOI: 10.1007/s002770000223.

86. Goldfeld M, Cohen I, Loberant N, et al. Littoral cell angioma of the spleen: appearance on sonography and CT. J Clin Ultrasound, 2002, 30(8): 510-513. DOI: 10.1002/jcu.10101.

87. Musgrave NJ, Williamson RM, O&#39, et al. Test and teach. Incidentally discovered splenic vascular lesion. Littoral cell angioma of the spleen. Pathology, 2002, 34(6): 579-581. DOI: 10.1080/0031302021000035983.

88. Johnson C, Goyal M, Kim B, et al. Littoral cell angioma. Clin Imaging, 2007, 31(1): 27-31. DOI: 10.1016/j.clinimag.2006.09.021.

89. Chang MK, Singh CS, Gupta R, et al. Extramedullary hemopoiesis with littoral cell angioma involving main and accessory spleens. Ann Hematol, 2007, 86(9): 695-696. DOI: 10.1007/s00277-007-0311-9.

90. Chatelain D, Bonte H, Guillevin L, et al. Small solitary littoral cell angioma associated with splenic marginal zone lymphoma and villous lymphocyte leukaemia in a patient with hepatitis C infection. Histopathology, 2002, 41(5): 473-475. DOI: 10.1046/j.1365-2559.2002.14313.x.

91. Michal M, Skálová A, Fakan F, et al. Littoral cell angioma of the spleen. A case report with ultrastructural and immunohistochemical observations. Zentralbl Pathol, 1993, 139(4-5): 361-365.

92. Barshack I, Perelman M, Many A, et al. Littoral cell angioma: a vascular tumor mimicking a solid tumor on a Tc-99m-red blood cell spleen scan. Isr J Med Sci, 1997, 33(10): 677-680.

93. Parrens M, Nouts C, Belleanne G, et al. Littoral cell angioma: a rare vascular splenic tumor. Ann Pathol, 1998, 18(6): 484-487.

94. Chan KY, Naqiyah I, Norlia A. Vascular tumours disguising as solid organ injuries on computed tomography: a report of two cases. Nepal Med Coll J, 2004, 6(2): 152-153.

95. Erçin C, Gürbüz Y, Hacihanefioğlu A, et al. Multiple littoral cell angioma of the spleen in a case of myelodysplastic syndrome. Hematology, 2005, 10(2): 141-144. DOI: 10.1080/10245330400026121.

96. Dema A, Tăban S, Cornianu M, et al. An unusual splenic tumor. Littoral cell angioma. Chirurgia (Bucur), 2007, 102(6): 739-743.

97. Maternini M, Misani M, Vanzati A, et al. Extramedullary hemopoiesis and littoral cell angioma of the spleen: our experience and review. Hepatogastroenterology, 2012, 59(118): 1789-1793. DOI: 10.5754/hge10810.

98. Mokhtari N, Hamidian Jahromi A, Dela Cruz N, et al. Littoral cell angioma: review of the literature and case report. J La State Med Soc, 2013, 165(6): 329-333.

99. Cai YQ, Wang X, Ran X, et al. Laparoscopic splenectomy for splenic littoral cell angioma. World J Gastroenterol, 2015, 21(21): 6660-6664. DOI: 10.3748/ wjg.v21.i21.6660.

100. Lyu S, He Q. Huge littoral cell angioma of the spleen: a case report. J Nippon Med Sch, 2019, 86(3): 179-182. DOI: 10.1272/jnms.JNMS.2019_86-307.

101. Li MJ, Zhou X, Cao JY, et al. Laparoscopic splenectomy for littoral cell angioma of the spleen: a case report. Medicine (Baltimore), 2019, 98(11): e14825. DOI: 10.1097/MD.0000000000014825.

102. Liu D, Chen Z, Wang T, et al. Littoral-cell angioma of the spleen: a case report. Cancer Biol Med, 2017, 14(2):194-195. DOI: 10.20892/ j.issn.2095-3941.2016.0094.

103. Gao C, Li YC, Xiao XM, et al. Littoral cell angioma in the spleen. Br J Hosp Med (Lond), 2015, 76(1): 55. DOI: 10.12968/hmed.2015.76.1.55.

104. Du J, Shen Q, Yin H, et al. Littoral cell angioma of the spleen: report of three cases and literature review. Int J Clin Exp Pathol, 2015, 8(7): 8516-8520.

105. Zhang YH, Liu LM, Wang WP, et al. Littoral cell angioma of the spleen: sonographic-pathologic comparison. J Ultrasound Med, 2013, 32(4): 691-697. DOI: 10.7863/jum.2013.32.4.691.

106. Liang W, Lu J, Qin M, et al. Littoral cell angioma mimicking hepatic tumor. Acta Radiol Short Rep, 2012, 1(6). DOI: 10.1258/arsr.2012.120031.

107. Hu ZQ, A YJ, Sun QM, et al. The splenic Littoral cell angioma in China: a case report and review. World J Surg Oncol, 2011, 9: 168. DOI: 10.1186/ 1477-7819-9-168.

108. Lin XY, Li JM, Wang QX, et al. Littoral cell angioma of the spleen: report of three cases and a review of the literature. Chin Med J (Engl), 2011, 124(20): 3423-3426.

109. Wang YJ, Li F, Cao F, et al. Littoral cell angioma of the spleen. Asian J Surg, 2009, 32(3): 167-171. DOI: 10.1016/S1015-9584(09)60389-4.

110. Cheng SP, Yang TL, Chen BF, et al. Image of the month. Littoral cell angioma. Arch Surg, 2005, 140(11): 1127-1128. DOI: 10.1001/archsurg.140.11.1127-a.

111. Lin CH, Yu JC, Shih ML, et al. Littoral cell angioma of the spleen in a patient with hepatocellular carcinoma. J Formos Med Assoc, 2005, 104(4): 282-285.

112. Qu ZB, Liu LX, Wu LF, et al. Multiple littoral cell angioma of the spleen: a case report and review of the literature. Onkologie, 2007, 30(5): 256-258. DOI: 10.1159/000101010.

113. Dong wei , Li feibo , Kang qingsong , et al. 6 cases of diagnosis and treatment of littoral cell angioma. Zhejiang Med J. 2019,41(15): 1653-1654.

114. Zhang Ying, Li Yunlong, Huang Deli, et al. Two-case-reviews with a rare spleen tumor with anemia and platelets . Lab Med Clin. 2017, 14(18):2751-2753.

115. Sun Chengming, Li Guanghao, Zhang Yubao. Experience in the diagnosis and treatment of 61 cases of spleen tumor. Mod Diagn Treat. 2016, 27(08): 1462-1464.

116. Gao feng, Wang Lifeng, Mei huanlin, et al. An example of littoral cell angioma. Chin J Surg. 2005, (09): 620-621.

117. Shi Da, Zhang Xuchang, ZhangXuHui. 26 cases of preoperative MSCT spleen tumor diagnosis analysis. Chinese Journal of New Clinical Medicine, 2019, 12(08):883-886.

118. Liu Hailong, Liu Min, Liu Yan, et al. The image of littoral cell angioma. Chin J Radiol. 2013, (05): 440-443.

119. Li Qing, Wang Linlin, Zhou xiaoqiu, et al. one case of spontaneous rupture of Littoral cell angioma. J Binzhou Med Coll. 2015, 38(05): 395-397.

120. Deng Liping, Yan Minjian, Zhang Shi Zheng. Littoral cell angioma: image performance and pathological control. Chin J Med Computed Imaging. 2008, (05): 415-419.

121. Li Hongwei, Wang Hua. An example of littoral cell angioma and reviewed in literature. Cancer Res Clin. 2013, 25(12): 838-839.

122. Wang Shunli, Lu Xiaoyu, Yin Yulei, et al. A report and literature review on a case of littoral cell angioma. Journal of Diagnostics Concepts＆Practice, 2010, 9(02): 173-176.

123. Cao Feng , Wang Yajun, Li Fei, et al. An example of Littoral cell angioma. Chin J Surg. 2008, (10): 727.

124. Yuan XiaoDong, Zhang Jing. An example of littoral cell angioma. Chin J Clin (Electron. Ed). 2010, 4(10):2054-2055.

125. Yu Min, Li Xiaoqiang, Huang Fang, et al. Clinical pathological analysis of littoral cell angioma. Chin J Clin Exp Pathol. 2016, 32(01): 91-93.

126. Zhang Jun, Huang Yuanquan, Huang Wenjie, et al. Diagnosis and treatment of nine cases of littoral cell angioma. Chin J Dig Surg. 2017, 23(10): 707-709.

127. Bian Yuhai, Shen Zhiyong, Mi Xingzhi, et al. Two cases of littoral cell angioma. Chin J Dig Surg. 2008, (03): 232-234.

128. Huang Guoquan, Wang Jianwen, Liu Bin, et al. Multi-layer helical CT image features of littoral cell angioma: 5 cases are reported. Chinese Journal of CT and MRI, 2014, 12(04): 107-110.

129. He Jian, Shen Jian, Zhou Wei, et al. Littoral cell angioma CT and MRI manifestations with pathological control. J Med Imaging. 2015, 25(03): 489-491.

130. Chen Jianyue. Pathological analysis of 3 cases of littoral cell angioma. Zhejiang Pract Med. 2007, (03): 177-179.

131. Hu Wei, Shi Kirin. Pathological analysis of 6 cases of littoral cell angioma. J Chin Oncol. 2011, 17(04): 311-312.

132. Wang Junyan, Sun Yanbao. Imaging of littoral cell angioma. J Med Imaging. 2017, 27(02): 284-295.

133. Wang Yanyan, Yang Jihu, Yu Lin, et al. Spiral CT and MRI performance of Littoral cell angioma. Chin J Primary Med Pharm. 2015, 22(1): 26-28. DOI: 10.3760/ cma.j.issn.1008-6706.2015.01.009.

134. Chen Qin, Shao Liming. Clinical and pathological analysis of littoral cell angioma. Chin J Dig Surg. 2009, 24(9): 765-766. DOI: 10.3760/cma. j.issn.1007-631X.2009.09.029.

135. Lu Jingjing, Liang Wenhua, Zhong Dingrong, etc. Clinical, pathological and imaging manifestations of littoral cell angioma. Chin J Med Imaging. 2012, 20(5): 368-371. DOI: 10.3969/j.issn.1005-5185.2012.05.013.

136. Pan Jiaohai, Liang Xiaochao. Multi-layer helical CT and MRI diagnosis of littoral cell angioma. Chin J Dig Surg. 2013, 28(11): 896-897.

137. Xue Honghong, Shen Juan, Bai Baoyan, et al. Ultrasound angiography and pathological control analysis of littoral cell angioma. J Med Imaging. 2017. 27(03): 489-491.

138. Ding Ying, Zeng Mengsu, Rao Shengxiang, et al. MRI manifestations of littoral cell angioma and control analysis with pathology. Radiologic Pract. 2012, 27(07): 761-764.

139. Li Ling, Xu Qing. CT manifestations of Littoral cell angioma (with 4 cases of reports and literature review). J Pract Radiol. 2016, 32(7): 1154-1155. DOI: 10.3969/ j.issn.1002-1671.2016.07.046.

140. Zhang Zhongshu, Zhang Song, Yan Xiaohang, et al. Relationship between hemangioma CT and MR image representation and pathological results of littoral cell angioma. Chin J Difficult Complicated Cases. 2018, 17(12): 1380-1383. DOI: 10.3969/j.issn.1671-6450.2018.12.019.

141. Zhang Hongqin. Pathological analysis of 5 cases of littoral cell angioma. Acta Acad Med Nantong. 2010, 30(2): 96-97. DOI: 10.3969/j.issn.1674-7887.2010.02.006.

142. Jing Meng, Wang Anqun, Yang Yonghong, et al. Clinical pathological analysis of 2 cases of littoral cell angioma. Practical Journal of Clinical Medicine, 2018, 15(01): 217-218.

143. Wu Shuo, Cheng Nianlan, LI Bangguo, et al. Clinical pathological analysis of 2 cases of littoral cell angioma. J Pract Radiol. 2019, 35(3): 506-507. DOI: 10.3969/j.issn.1002-1671.2019.03.045.

144. Wang Yijing, Sun Yanqiu, Li Yan, et al. 2 cases of Littoral cell angioma. J Pract Radiol. 2019, 35(5): 854-855. DOI: 10.3969/j.issn.1002-1671.2019.05.048.

145. Xiao Hai, Liu Sijun, Yang Qingchun. Clinical pathology analysis of 1 case of littoral cell angioma. J Ganan Med Coll. 2011, 31(02): 238-239.

146. Li Duofu, Yan Xuanxuan, Cao Nong. 1 case of littoral cell angioma was reported and reviewed in literature. Chin J Med Imaging. 2017, 33(19): 3048-3050.

147. Wang Boyuan, Chen Zejun. 1 case of CT and MRI performance of littoral cell angioma. Chin J Med Imaging. 2013, 21(01): 46-47.

148. Luo Xiaoqin, Chen Guangbin, Cao Yang. 1 case of littoral cell angioma. J Med Imaging. 2018, 28(07): 1064-1068.

149. Han Songbo, Xu Hui, Jiang Pengcheng, et al. 1 case of Littoral cell angioma. Chin J Med Imaging Technol. 2010, 26(10): 1813

150. Yang Qingge, Jindi, Zhang Wei. 1 case of Littoral cell angioma. People's Military Surgeon, 2015, 58(3): 353.

151. Yang Qianpeng, Liu Nijun, Wang Minru, et al. 1 case of Littoral cell angioma. Chin J Med Imaging Technol. 2017, 33(11): 1757. DOI: 10.13929/j.1003-3289. 201702103.

152. Li Guimei, Le Mei zhao, Gao Hong. Clinical pathology of 3 cases of littoral cell angioma. Chin J Diagnostic Pathol. 2011, 18(2): 110-112. DOI: 10.3969/j.issn. 1007-8096.2011.02.010.

153. Chen Hongmei, Kong Zhaoru, Zhang Guoqiong. 1 case of Littoral cell angioma. Chin. J Clin Exp Pathol. 2001, 17(3): 197. DOI: 10.3969/j.issn.1001-7399. 2001.03.029.

154. Zhang Da, Zheng Jianming. Discussion on the diagnosis of littoral cell angioma (with a case report). Acad J Second Mil Med Univ. 2004, 25(8): 904-905. DOI: 10.3321/j.issn:0258-879X.2004.08.030.

155. Lin Yali, Xu Xianzhao, Jiang Xiaoxuan. An example of littoral cell angioma and literature review. Hainan Med J. 2015, (16): 2481-2482. DOI: 10.3969/j.issn. 1003-6350.2015.16.0899.

156. Jiang Huijun, Yu Jie, Yu Ping. A report and literature review on a case of littoral cell angioma. Chin J Surg Oncol. 2011, 3(6):321-324,340. DOI: 10.3969/j.issn.1674-4136.2011.06.001.

157. Han Kaize, Wang Shupeng, Guan Qingchun, et al. A case of littoral cell angioma. J. Hepatopancreatobiliary Surg. 2018, 30(5): 434-435. DOI: 10.11952/j.issn. 1007-1954.2018.05.022.

158. Yuan Chunhui, Xiu Dianrong, Zhang Tonglin. An example of littoral cell angioma. Chin J Clin Exp Pathol. 2013, 19(4): 300: 320. DOI: 10.3760/cma. j.issn.1007-8118.2013.04.016.

159. Wang Min. Clinical pathology of littoral cell angioma. China Medicine and Pharmacy. 2014, (12): 167-169,200.

160. Zhang Lihua, Chen Wei. Clinical pathology analysis of littoral cell angioma. Chin J Clin Exp Pathol. 2011, 27(1): 99-100. DOI: 10.3969/j.issn. 1001-7399.2011.01.026.

161. Gao Jinli. Clinicopathological analysis of littoral cell angioma. Chin J Clin (Electron. Ed). 2012, 6(19): 6080-6081. DOI: 10.3877/cma.j.issn.1674-0785.2012. 19.088.

162. Lu Shaocheng, Zhou Xiang, Pan Bing, et al. Two cases report of littoral cell angioma and literature review. International Journal of Surgery. 2018, 45(10): 688-690. DOI: 10.3760/cma.j.issn.1673-4203.2018.10.010.

163. Qing Shenglan, Liu Zhongliang, Chen Yuxiang, et al. Diagnosis and treatment of littoral cell angioma. Journal of Cancer Control and Treatment. 2008, (03): 298-299.

164. Wang Tao, Ju Lingyan, Jiang Zehui. Clinicopathological analysis of littoral cell angioma (report of 2 cases). Journal of Basic and Clinical Oncology. 2009, 22(05): 423-425.

165. Cao Zhong, Wei Jianguo, Cen Hongbing, et al. 13 cases of littoral cell angioma. J Peking Univ Health Sci. 2017, 49(03): 495-500.

166. Shu Meiling, Xia Sunwen. Clinicopathological analysis of 3 cases of littoral cell angioma. Current Physician. 2012, 18(11): 86-87.

167. Hao Hua, Xu Fen, Wu Liqing, et al. Clinicopathological observation of 2 cases of Littoral cell angioma. Chin J Diagnostic Pathol. 2016, 23(11): 870-874.

168. Fang Wei, Wang Luxiang, Fan Qinhe, et al. Clinicopathological observation of 2 cases of littoral cell angioma. Chin J Diagnostic Pathol. 2011, 18(01): 56-59.

169. Jian Xinmei, Fan Guangming, Song Lingling. Imaging findings of 2 cases of littoral cell angioma. J Shanxi Med Univ. 2018, 49(09): 1134-1136.

170. Ji Xiang, Yu Jianping, Shi Xin, et al. A case of littoral cell angioma and literature review. J Mod Oncol. 2018, 26(11): 1757-1759.

171. Hao Yanyong, Wu Weiguo, Wu Yan, et al. A case of littoral cell angioma and literature review. Chinese Journal of Laboratory Diagnosis, 2013, 17(06):1158-1159.

172. Zhang Hongkai, Deng Hongbing, Yi Yongfen, et al. A case of littoral cell angioma and literature review. Chin J Clin Exp Pat. 2004, (04): 503-504.

173. Jia feng, Jin Kai, Liu Xiangliang, et al. A case report of littoral cell angioma. Chin J Clin Hepatol. 2017, 33(09): 1787-1788.

174. Yang Daohua, Qiu Chenmin, Liu Di, et al. One case report of littoral cell angioma. Chin J Diagnostic Pathol. 2008, 15(06): 488-489.

175. Zhang Lihua, Sun Yingchao, Chen Ying. One case report of littoral cell angioma. Chin J Diagnostic Pathol. 2008, (02): 147-148.

176. Sun Xiaobo, Jiang Lisheng, Li Fuyu, et al. A case of littoral cell angioma. Journal of Digestive Surgery, 2006, (03): 174-226.

177. Wu Chaojun, Wei Dayou. A case of littoral cell angioma. Chin J Ultrasonography. 2001, (07): 28.

178. Liu Shasta, Shen Xingfu, Zhao zing, et al. A case of littoral cell angioma. J Chengde Med Coll. 2013, 30(04): 343-344.

179. Li ruixi, Cao mingbo, Xu jianliang, et al. A case of littoral cell angioma. Chin J Gen Surg. 2018, 33(11): 974-975.

180. Liu Dongming, Zhou Hongzhou, Chen Xing, et al. A case of littoral cell angioma. Chin J Clin Oncol. 2016, 43(19): 877.

181. Li Peilin, Wen Xianhua, Chen Yuantao, et al. A case of littoral cell angioma treated by laparoscopy. Chinese Journal of Laparoscopic Surgery (Electro Ed). 2017, 10(03): 175-176.

182. Yam Zhibo, Wu Xiaojuan, Zhan Hanxiang, et al. Laparoscopic splenectomy for the treatment of Littoral cell angioma: report of 3 cases and literature review. Journal of Laparoscopic Surgery. 2017, 22(08): 588-591.

183. Chen Bo, Ding Shanshan, Liu Shan, et al. One case report of littoral cell angioma with obvious extramedullary hematopoiesis. Chin J Diagnostic Pathol. 2013, 20(03): 176-177.

184. Jiang Chunjuan, Tao Jingjing, Tang Ping, et al. CT diagnosis of one case of Littoral cell angioma. Chinese Journal of Anatomy and Clinics. 2015, 20(04): 375-376.

185. Wang Chao, Tian Ying, Cai Wenjing, et al. Littoral cell angioma with hemorrhage-film reading window in 2019. Anhui Yixue. 2019, 40(09): 1076-1077.

186. Lu Tao, Pu Hong, Chen Guangwen, et al. CT manifestations and differential diagnosis of primary vascular tumors of the spleen. J Clin Radiol. 2019, 38(07): 1244-1248.

187. Feng Wei, Zhu Jiasheng, Yao Kai, et al. A case of littoral cell angioma. Chin J Gen Surg. 2012, 21(12): 1605-1606.

188. Xie Shanshan, Zhang Yong, Cheng Jingliang. A case of MR manifestations of splenic sinus bank cell hemangioma. Chin J Magn Reson Imaging. 2020, 11(06): 453-454.

189. Liu Yanjie, Fan Zhengjun, Xue Jianfeng, et al. Diagnosis of 1 case of splenic sinus bank cell hemangioma. J Zhengzhou Univ Med Sci. 2011, 46(06): 949-951.
